# Transcriptome‐wide gene expression outlier analysis pinpoints therapeutic vulnerabilities in colorectal cancer

**DOI:** 10.1002/1878-0261.13622

**Published:** 2024-03-11

**Authors:** Elisa Mariella, Gaia Grasso, Martina Miotto, Kristi Buzo, Nicole Megan Reilly, Pietro Andrei, Pietro Paolo Vitiello, Giovanni Crisafulli, Sabrina Arena, Giuseppe Rospo, Giorgio Corti, Annalisa Lorenzato, Carlotta Cancelliere, Ludovic Barault, Giulia Gionfriddo, Michael Linnebacher, Mariangela Russo, Federica Di Nicolantonio, Alberto Bardelli

**Affiliations:** ^1^ Department of Oncology, Molecular Biotechnology Center University of Torino Italy; ^2^ IFOM ETS, The AIRC Institute of Molecular Oncology Milan Italy; ^3^ Department of Oncology University of Torino Candiolo (TO) Italy; ^4^ Candiolo Cancer Institute FPO‐IRCCS Candiolo (TO) Italy; ^5^ Clinic of General Surgery, Molecular Oncology and Immunotherapy University of Rostock Germany; ^6^ Present address: Boehringer Ingelheim RCV GmbH & Co KG Vienna Austria

**Keywords:** biomarkers, colorectal cancer, drug targets, gene expression outliers

## Abstract

Multiple strategies are continuously being explored to expand the drug target repertoire in solid tumors. We devised a novel computational workflow for transcriptome‐wide gene expression outlier analysis that allows the systematic identification of both overexpression and underexpression events in cancer cells. Here, it was applied to expression values obtained through RNA sequencing in 226 colorectal cancer (CRC) cell lines that were also characterized by whole‐exome sequencing and microarray‐based DNA methylation profiling. We found cell models displaying an abnormally high or low expression level for 3533 and 965 genes, respectively. Gene expression abnormalities that have been previously associated with clinically relevant features of CRC cell lines were confirmed. Moreover, by integrating multi‐omics data, we identified both genetic and epigenetic alternations underlying outlier expression values. Importantly, our atlas of CRC gene expression outliers can guide the discovery of novel drug targets and biomarkers. As a proof of concept, we found that CRC cell lines lacking expression of the *MTAP* gene are sensitive to treatment with a PRMT5‐MTA inhibitor (MRTX1719). Finally, other tumor types may also benefit from this approach.

## Graphical abstract

Multi‐omics data were obtained for 226 CRC cell lines. Extreme positive and negative gene expression outliers were identified from RNA‐seq data using a novel computational workflow. Gene expression abnormalities were subsequently associated with (epi)genetic alterations. Dataset exploration can guide the identification of drug targets and biomarkers through gene annotation, based on publicly available repositories, and drug screening assays.

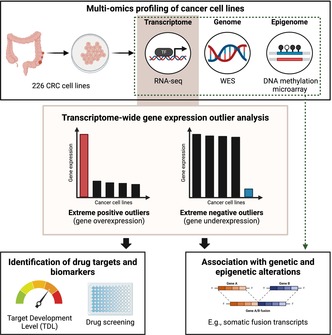

**Abbreviations**
CGI: CpG islands
CIMP: CpG island methylator phenotype
CMS: consensus molecular subtypes
CNV: copy number variation
CRIS: CRC intrinsic subtypes
FDR: false discovery rate
IQR: interquartile range
mCRC: metastatic colorectal cancer
MMR: mismatch repair
MSI: microsatellite instability
MSS: microsatellite stability
PDX: patient‐derived xenograft
Q1: first quartile
Q3: third quartile
RNA‐seq: RNA sequencing
SNV: single‐nucleotide variant
STR: short tandem repeats
TDL: target development level
TMZ: temozolomide
WES: whole‐exome sequencing

## 1 Introduction

The advent of next‐generation sequencing technologies has revolutionized the field of oncology, supporting the introduction of precision medicine approaches [1, 2]. In this context, the identification and characterization of somatic mutations has led to multiple avenues. First, it has driven the design of inhibitors targeting mutated proteins in oncogenic signaling pathways, thus leading to clinically effective drugs such as EGFR and BRAF inhibitors in lung cancers and melanomas [3, 4]. Second, it has been essential for the identification of biomarkers in signaling pathways downstream of targeted proteins [5, 6]. However, in addition to mutations, other molecular alterations have proved to be useful for identifying clinically relevant features of human tumors and cancer cell‐specific vulnerabilities [1, 2]. A paradigmatic example is *ERBB2* gene amplification which is found at variable frequency in multiple tumor types, such as breast and colorectal cancer, where it leads to sensitivity to HER2 inhibition [7, 8]. In a similar way, TRK inhibitors have been approved in a tissue‐agnostic way for solid tumors that harbor genetic rearrangements involving NTRK genes [9]. Importantly, in both cases, a genetic alteration leads to gene overexpression that results in aberrant protein activity. On the other hand, the expression of multiple genes is lost during tumor evolution due to homozygous deletion or epigenetic silencing [10, 11]. This can determine the onset of clinically relevant features. For example, *MLH1* silencing occurs in sporadic colorectal cancer (CRC) due to promoter hypermethylation, and it induces microsatellite instability [12]. Similarly, promoter hypermethylation may determine loss of *MGMT* expression, thus conferring sensitivity to temozolomide (TMZ) that is an essential component of the current standard protocol for glioblastoma treatment [13]. Intriguingly, gene silencing can also be associated with synthetic lethal targets. For instance, cancer cells that are *RAD51C*‐deficient are more sensitive to PARP inhibition than cells with normal *RAD51C* expression levels [14]. Finally, in addition to vulnerabilities related to overexpression or silencing of individual genes, the concept of transcriptional addiction has been recently proposed to describe dysregulated gene expression programs that create vulnerabilities not predicted by genetic changes [15]. Overall, these examples highlight that methods to systematically identify aberrant expression levels in cancer cells may be helpful to rapidly find genes that deserve an in‐depth characterization, with the final aim of pinpointing drug targets and biomarkers.

Samples that exhibit an abnormally high or low expression level for a particular gene are usually referred to as “gene expression outliers” [16]. Several studies have exploited the identification of gene expression outliers to establish which tumor samples or preclinical models were characterized by overexpression of druggable genes [17, 18, 19]. For example, a previous study by our group looked for expression outliers across 151 CRC cell lines to identify kinase genes driving resistance to EGFR blockade and found that CRC cell lines were functionally and pharmacologically addicted to overexpressed kinases [17]. Although the results of these studies were conceptually similar, different methods were used for defining gene expression outliers. In some cases, outliers were identified based on the deviation of each sample from the median expression using arbitrary thresholds. Alternatively, the popular Tukey's rule was applied. In this case, outlier calling is based on gene‐specific thresholds since, given the characteristic distribution of expression values of each single gene, outliers are found as values more than 1.5 interquartile ranges (IQR) above the third quartile (Q3) or below the first quartile (Q1). Differently, Kothari et al. focused on the comparison of the expression levels of multiple kinase genes in each individual sample, that is, they looked for “outlier genes in individual samples” rather than “samples displaying outlier expression of candidate genes” [20]. Importantly, they found that cancer cell lines are sensitive to the inhibition of outlier kinases. Therefore, despite methodological differences, their conclusion was in line with that of the studies that looked for outlier samples.

We reasoned that, although successful, the scope of previous studies was limited to restricted gene subsets, most often kinases. Therefore, we hypothesized that additional vulnerabilities could be uncovered by extending the gene expression outlier analysis to the entire coding genome and by concomitantly profiling gene underexpression and overexpression events. From now on, in this work we will refer to samples showing overexpression of an individual gene as “positive outliers,” while “negative outliers” are samples characterized by the underexpression of a single gene.

In the present study, we applied this concept to gene expression data from a collection of 226 CRC cell lines. CRC is the fourth most common malignancy and the third leading cause of cancer‐related death worldwide [21]. While it is a curable disease at early, localized stages, around 20% of patients are diagnosed at the metastatic stage and 40% develop metastasis after initial treatment of localized disease [22]. Unfortunately, the high inter‐patient heterogeneity and other distinctive features of CRC have made the development of targeted therapies particularly difficult for metastatic CRC patients [23]. Nevertheless, in the last decade, studies on CRC preclinical models, recapitulating the genomic, phenotypic, and drug response diversity that is observed in patients, have stimulated the design of successful clinical trials testing inhibitors of oncogenic drivers found in small but clinically relevant fractions of CRC patients [23]. Further exploration of existing and novel preclinical models may reveal additional therapeutic hits, aiming to offer targeted therapies to an ever‐increasing number of CRC patients.

## 2 Materials and methods

### 2.1 Cell cultures

The official names of the CRC cell lines used in the study and Research Resource Identifiers (RRIDs), as available in the ExPASy Cellosaurus database, are reported in Table S1. The source of each cell line and a list of relevant references for each of them are also indicated. Cell cultures were supplemented with FBS 10% 2 mm l‐glutamine, antibiotics (100 U·mL^−1^ penicillin and 100 mg·mL^−1^ streptomycin) and grown in a 37 °C and 5% CO_2_ air incubator. Cell cultures were routinely screened for the absence of Mycoplasma contamination using the Venor^®^ GeM Classic kit (Minerva Biolabs, Berlin, Deutschland). The identity of each cell line was checked no more than 3 months before performing the experiments using the PowerPlex^®^ 16 HS System (Promega, Madison, WI, USA), through Short Tandem Repeats (STR) tests at 16 different loci (D5S818, D13S317, D7S820, D16S539, D21S11, vWA, TH01, TPOX, CSF1PO, D18S51, D3S1358, D8S1179, FGA, Penta D, Penta E, and amelogenin). Amplicons from multiplex PCRs were separated by capillary electrophoresis (3730 DNA Analyzer; Applied Biosystems, Thermo Fisher Scientific, Waltham, MA, USA) and analyzed using genemapper v.3.7 software (Life Technologies, Thermo Fisher Scientific). The STR profile of each CRC cell line is reported in Table S2.

### 2.2 Microsatellite status

The microsatellite status of 224 out of 226 CRC cell lines was assessed with the MSI Analysis System kit (Promega). The analysis requires a multiplex amplification of seven markers including five mononucleotide repeat markers (BAT‐25, BAT‐26, NR‐21, NR‐24, and MONO‐27) and two pentanucleotide repeat markers (Penta C and Penta D). The amplification products were analyzed by capillary electrophoresis in a single injection (3730 DNA Analyzer, ABI capillary electrophoresis system; Applied Biosystems). Then, the results were analyzed using genemapper v.5.0 software. The microsatellite status of the remaining two CRC cell lines (IRCC22_XL and HROC222_T1_M2) was determined based on whole‐exome sequencing (WES) data using MSIsensor‐pro [24]. The microsatellite status of each CRC cell line is reported in Table S1 as MSS (microsatellite stability) or MSI (microsatellite instability).

### 2.3 Genomic DNA extraction and sequencing

Whole‐exome sequencing data were available for 149 out of 226 CRC cell lines from previous studies (Table S1). WES data were therefore obtained for the remaining 77 CRC cell lines. In addition, from a previous study we retrieved WES data from matched normal samples obtained from the same patients from which 25 out of 226 CRC cell lines were derived [19]. Genomic DNA (gDNA) was extracted from both CRC cell lines and normal samples using Maxwell^®^ RSC Blood DNA kit (AS1400; Promega). Library preparation, sequencing, and data demultiplexing were outsourced. Final DNA libraries were sequenced as paired‐end 100 bp reads.

### 2.4 RNA extraction and sequencing

RNA sequencing (RNA‐seq) data were available for 140 out of 226 CRC cell lines from previous studies (Table S1). RNA‐seq data were therefore obtained for the remaining 86 CRC cell lines. Total RNA was extracted using Maxwell^®^ RSC miRNA Tissue Kit (AS1460; Promega). The quantification of RNA was performed by Thermo Scientific Nanodrop 1000 (Agilent, Santa Clara, CA, USA) and Qubit 3.0 Fluorometer (Life Technologies). RNA integrity was evaluated with the Agilent 2100 Bioanalyzer using the Agilent RNA 6000 Nano Kit. Total RNA (800 ng) with RNA integrity number (RIN) score between 9 and 10 was used as input to the Illumina TruSeq RNA Sample Prep Kit v2‐Set B (48Rxn), according to the manufacturer's protocol. After library preparation, sequencing was performed on NextSeq500 to get single‐end 150 bp reads.

### 2.5 DNA methylation microarray experiments

DNA methylation data were previously obtained for 146 CRC cell lines using the Infinium HumanMethylation450 BeadChip array [25]. The DNA methylation profile of 80 additional CRC cell lines was obtained for this work with the Infinium MethylationEPIC BeadChip microarray, which allowed for over 850 000 methylation sites to be quantitatively interrogated across the genome at single‐nucleotide resolution. Preparation and processing of DNA samples were carried out using the Illumina Infinium HD Assay Methylation Protocol Guide. Briefly, DNA samples (500 ng) were treated with sodium bisulfite using the Zymo EZ‐96 DNA Methylation‐Lightning Kit (Zymo Research, Irvine, CA, USA). The bisulfite‐converted DNA was used for amplification, fragmentation, precipitation, resuspension, and hybridization on BeadChips. Next, single‐base extension and staining were performed following the Infinium HD Methylation automated workflow. Finally, the iscan software analysis system (Illumina, San Diego, CA, USA) was used for signal acquisition and analysis.

### 2.6 Identification of somatic mutations

Whole‐exome sequencing reads were aligned to human genome version 38 (hg38) using bwa‐mem algorithm [26]. PCR and optical duplicates were annotated and ignored in the subsequent processing. Then, different modules previously developed by our group were exploited for identification of single‐nucleotide variants (SNVs) and indels (IDEA workflow) [27]. Bases with Phred quality score lower than 20 (Q20) were discarded. In addition, only SNVs supported by at least 10 altered reads and whose mutant allele frequency was higher than 10% were selected. Furthermore, common SNPs from dbSNP version 151 were used to exclude germinal variants, and synonymous mutations were ignored in the determination of gene mutational status. Similarly, only indels supported by at least 10 altered reads and whose mutant allele frequency was higher than 10% were considered. *KRAS* (G12, G13, A59, Q61, K117, and A146), *NRAS* (G12, G13, and Q61), and *BRAF* (V600E) hotspots were considered in the annotation of the mutational status of these genes. In parallel, hotspot mutations in *TP53* (R175, G245, R248, R249, R273, and R282), nonsense mutations, and frameshift indels were considered as mutations that likely determine protein loss of function when annotating *TP53* and *APC* mutational status.

### 2.7 Copy number analysis

We adapted a previously described strategy to deal with the fact that WES data from matched normal samples are not available for the majority of CRC cell lines [27]. In each CRC cell line, gene copy number (CN) values were computed as the ratio between the median read depth of each gene and the median read depth of all genes using GENCODE v33 as gene annotation. A principal component analysis (PCA) of gene CN values revealed three different sample clusters that were confirmed through the application of *k*‐means clustering on the first 10 principal components. Based on these findings, a “metanormal” was created for each sample cluster taking for each gene the median CN value across samples, and genes whose median CN value was lower than 0.1 in either one of the clusters were excluded from subsequent analysis (genes not captured by WES probes). Then, copy number variation (CNV) values were computed for every gene in every sample as the ratio between the gene CN value in the sample and the gene CN value in the corresponding metanormal. This corrects for systematic differences in sequencing depth among genes reflecting variability in the affinity of WES probes.

To validate the procedure, we exploited 25 CRC cell lines for which WES data from matched normal samples are available [19]. Importantly, when we compared log_2_‐transformed CNV values obtained using the matched normal or the metanormal, we usually found a high correlation (the median Pearson correlation coefficient was 0.78).

Previously, in tumor samples, we considered the gene copy number as altered when the log_2_‐transformed CNV values were higher than 1 or lower than −1 [28]. Here, more stringent thresholds were used to select only gene amplifications or homozygous deletions. In detail, a gene amplification was called when the log_2_‐transformed CNV value was higher than 2, while a gene deletion was identified in samples whose log_2_‐transformed CNV value was lower than −2.

### 2.8 Gene expression values and molecular subtypes

RNA‐seq reads were aligned to hg38 using the splice‐aware mapsplice aligner [29]. Output BAM files were processed using ubu sam‐xlate and sam‐filter to translate genomic coordinates into transcriptomic ones and remove reads with indels, large inserts, and zero mapping quality before proceeding with transcript and gene quantification using RSEM [30] and GENCODE v33 as gene annotation. Then, starting from RSEM genes results (expected counts and effective lengths), we computed robust FPKM values exploiting the tximport R Bioconductor package and the fpkm function included in the deseq2 R Bioconductor package [31]. The resulting gene expression matrix was subsequently annotated with gene names from the GENCODE annotation file and filtered by applying the following criteria: (a) genes on chrM or chrY pseudoautosomal regions were removed; (b) genes with robust FPKM < 1 in all analyzed samples were considered not expressed and removed; and (c) only protein‐coding genes, as defined by the GENCODE annotation file, were selected for subsequent analysis.

In order to check RNA‐seq data quality with respect to the presence of CRC cell lines derived from the same individual (same STR profile), hierarchical clustering as implemented by the hclust R function was applied to log_2_‐transformed gene expression values using Pearson correlation coefficient to estimate sample‐wise distances and the ward.D2 agglomeration method. Furthermore, prediction of gene expression‐based subtypes, that is, consensus molecular subtypes (CMS) and CRC intrinsic subtypes (CRIS), were performed after log_2_ transformation of gene expression values using cmscaller and crisclassifier R packages with default parameters [32, 33]. In both cases, CRC cell lines that could not be confidently assigned to a single subtype (FDR > 5%) were labeled as NA (not available).

### 2.9 Identification of somatic fusion transcripts

RNA‐seq reads were processed using two publicly available tools (star‐fusion (v1.10.0) and fusioncatcher (v1.33)) to identify somatic fusion transcripts in CRC cell lines [34, 35]. star‐fusion predicted fusions were filtered based on the number of supporting reads (FFPM > 0.1 as suggested by the authors), and fusions thought to be red herrings (i.e., fusions that may not be relevant for cancer and potential false positives) were excluded based on star‐fusion automatic annotation. Then, to further control for the presence of artifacts, we required somatic fusion transcripts to be called also by fusioncatcher that was executed with default parameters. Finally, somatic fusion transcripts identified in CRC cell lines in which the amplification of the 3′ partner gene was also found were not considered in subsequent analysis, since amplification‐associated fusion transcripts likely are a by‐product of chromosomal instability, as previously reported [36].

### 2.10 DNA methylation levels and annotation of the CpG island methylator phenotype

HM450 and EPIC raw data (IDAT files) were processed with a similar workflow using the minfi R Bioconductor package and corresponding annotation packages [37]. In each sample, probes with detection *P*‐value > 0.05 were masked in the RGChannelSet. In addition, cross‐reactivity probes and probes matching SNPs at the target CpG site were filtered using previously published lists [38, 39]. Then, methylation and unmethylation signals were obtained for each CpG site using the preprocess Noob function, and β‐values were computed. Results obtained in all samples were subsequently merged in a single β‐matrix, thus keeping only CpG sites targeted by both HM450 and EPIC. Furthermore, CpG sites that are located on sex chromosomes or chrM were excluded.

For the classification of CRC cell lines with respect to CpG island methylator phenotype (CIMP), we referred to a previously published list of 318 probes that showed significantly higher DNA methylation levels in both CIMP‐H and CIMP‐L tumors compared to non‐CIMP tumors (CIMP‐associated probes) [40]. Starting from the β‐matrix obtained as described above, we selected the CIMP‐associated probes and excluded those containing missing values. Then, we performed unsupervised clustering using recursively partitioned mixture model (RPMM) with maxlevel = 2 and finally associated the resulting clusters to CIMP classes based on their median DNA methylation level (CIMP‐H, CIMP‐L, CIMP3, and CIMP4, for high to low median DNA methylation level).

### 2.11 Transcriptome‐wide gene expression outlier analysis in cancer cell lines

Our pipeline for the identification of transcriptome‐wide gene expression outliers relies on two different steps. As a starting point, the Tukey's method is applied to the log_2_‐transformed FPKM values of each single gene. In detail, the first (Q1) and the third (Q3) quartiles are obtained, and the interquartile range (IQR) is computed as Q3–Q1. Then, positive outliers are identified as samples whose log_2_‐transformed expression level is higher than Q3 + 1.5 × IQR. Similarly, negative outliers are identified as samples whose log_2_‐transformed expression level is lower than Q1–1.5 × IQR. In the second part of the pipeline, multiple filters are exploited to select the strongest gene expression alterations, assuming that they have a higher likelihood of being functionally relevant in CRC cells.

1. The furthest positive and negative outliers of each gene are selected. When multiple samples are identified as positive or negative outliers for the same gene, two groups of outliers can be distinguished. Specifically, the positive outliers can be subdivided into those that are nearest to the maximum expression value and those that are nearest to the highest expression value that is not an outlier. The positive outliers in the former group are the furthest positive outliers for that gene. Similarly, the furthest negative outliers of each gene are those whose distance from the minimum expression level is less than the distance from the lowest expression level that is not an outlier.
2. Positive and negative outliers for which the absolute value of the differential expression level is higher than 3 are selected. For each gene expression outlier, the differential expression level is computed as the log_2_ fold change with respect to the median gene expression using this formula: log_2_((FPKM+1)/(median + 1)).
3. Positive and negative outliers are further filtered based on the absolute expression level. Specifically, only positive outliers that have FPKM > 10 are selected, while negative outliers are retained if they have FPKM < 1.

We refer to the resulting gene expression outliers as “extreme positive outliers” and “extreme negative outliers” to distinguish them from positive and negative outliers initially obtained by applying the Tukey's method.

The described pipeline was applied to gene expression data from 226 CRC cell lines for which multi‐omics (WES, RNA‐seq, and DNA methylation microarray) data were available. We reasoned that the gene expression outlier analysis should be done separately for female and male samples in the case of genes on sex chromosomes. However, due to missing information on gender of individuals from which the cell lines were derived and since sex chromosomes are frequently lost in cancer cells [41], genes located on sex chromosomes were excluded from the gene expression outlier analysis.

### 2.12 Computation of outlier burden values in CRC cell lines

For each CRC cell line, we first considered the number of overexpressed genes (i.e., the genes for which it was selected as an “extreme positive outlier”) and the number of underexpressed genes (i.e., the genes for which it was selected as an “extreme negative outlier”). Then, these values were normalized with respect to the number of genes for which each CRC cell line could have been selected as an “extreme outlier” based on the filtering strategy described above. In detail, the “positive outlier burden” of each model is obtained by dividing the number of overexpressed genes by the number of genes that have FPKM > 10 and then multiplying the result by 1000. Similarly, the “negative outlier burden” of each model is obtained by dividing the number of underexpressed genes by the number of genes that have FPKM < 1 and then multiplying the result by 1000. The “positive outlier burden” and the “negative outlier burden” of each model are then summed to get the “total outlier burden.”

### 2.13 Identification of aberrantly expressed genes in CRC cell lines with the outlier in RNA‐seq finder (OUTRIDER) algorithm

OUTRIDER [42] was applied to gene expression data from 226 CRC cell lines, starting from RSEM gene counts. In detail, to allow a fair comparison between the different methods, OUTRIDER was used to analyze the expression profile only of those genes that have been considered in the transcriptome‐wide gene expression outlier analysis performed with our approach. The fitting of an autoencoder was used to automatically control for confounders that could have been present in the data. Since OUTRIDER assumes that samples are independent, in the case of CRC cell lines that are genetically identical, only one model for each group was selected and the others were masked during the autoencoder fitting. The optimal encoding dimension was automatically estimated and found to be *q* = 14 for this dataset. Outlier samples for each gene were identified as those whose adjusted *P*‐value was lower than 0.05, and then *z*‐scores were used to distinguish overexpressed and underexpressed genes.

### 2.14 Integration of gene expression outliers with promoter DNA methylation levels

We tested whether outlier gene expression values are associated with DNA hypermethylation or hypomethylation in promoter regions. First, we selected genes for which at least two CRC cell lines were identified as extreme positive or negative outliers, and from the β‐matrix we extracted the CpG sites that are located in the corresponding promoter region defined as 1500 bp upstream of transcription start site(s). Then, for each gene we compared the β‐values obtained for each CpG site within its promoter region between outliers and remaining samples by performing a Wilcoxon test, and FDR values were computed by applying the Benjamini–Hochberg method. Promoter CpG sites were considered differentially methylated between outlier and other samples when both FDR < 0.05 and a high difference in β‐values were observed. In the case of extreme positive outliers, the median β‐value must be > 0.80 in outlier samples and < 0.20 in other samples; on the contrary, in the case of extreme negative outliers, the median β‐value must be < 0.20 in outlier samples and > 0.80 in other samples. Furthermore, the analyzed CpG sites were annotated with respect to CpG islands (CGI). Starting from the University of California Santa Cruz (UCSC) annotation track for CpG islands in hg38, we segmented the human genome in CGI, CGI shores (i.e., 2 kb flanking a CGI on the left and on the right), CGI shelves (i.e., 2 kb flanking outwards a CGI shore), and open sea (i.e., the rest of the genome). This annotation was used to compare the genomic position of CpG sites that are differentially methylated with that of the non‐significant ones. Moreover, to classify genes for which we found a significant association between underexpression and promoter hypermethylation in extreme negative outliers in “associated with CIMP” and “not associated with CIMP,” we tested whether outlier samples were enriched for CIMP samples (i.e., CIMP‐H and CIMP‐L samples) by performing a hypergeometric test, and FDR values were computed by applying the Benjamini–Hochberg method. A significant association with the CIMP phenotype was called for genes for which FDR < 0.05 was obtained.

### 2.15 Enzymes, kinases, and target development‐level classification

The Swiss‐Prot database within the Universal Protein Resource Knowledgebase (UniProtKB) was downloaded in XML format on 17/11/2021 [43]. The XML file was then processed with a custom script to select human proteins with the following annotations if available: entry primary accession in UniProtKB, entry name in UniProtKB, recommended protein name, Enzyme Commission (EC) number(s), primary gene name, Ensembl gene ID(s), and Target Development Level (TDL). Swiss‐Prot UniProtKB retrieved the TDL classification of human proteins from the Pharos portal that provides access to data collected by NIH Illuminating the Druggable Genome (IDG) program [44]. Then, to focus on genes coding for enzymes, we selected Swiss‐Prot UniProtKB entries that were associated with EC numbers, meaning that they have catalytic activity, and we used the resulting list of genes to filter the extreme gene expression outliers. In addition, a further selection was performed considering an independent collection of 535 human kinases that were classified into different families, including both the eukaryotic protein kinase superfamily (TK, TKL, STE, CK1, AGC, CAMK, CMGC, other) and atypical kinases [45]. However, human kinases that according to Swiss‐Prot UniProtKB do not have detectable kinase activity were excluded (CAMKV, EPHB6, FAM20A, GASK1B, PRKY, ROR1, TRIB1).

### 2.16 Western blot

Whole protein lysates were isolated from cell lines by boiling in SDS buffer (50 mmol·L^−1^ Tris–HCl (pH 7.5), 150 mmol·L^−1^ NaCl, and 1% SDS) to extract total cellular proteins. The lysates were quantified by the BCA Protein Assay Reagent kit (Thermo Fisher Scientific) and prepared for western blot using LDS and Reducing Agent (Invitrogen, Thermo Fisher Scientific). Western blot analysis was performed with Enhanced Chemiluminescence System (Cytiva, Marlborough, MA, USA) and peroxidase‐conjugated secondary antibodies (Cytiva). The following primary antibodies were used for western blotting: anti‐MTAP (Santa Cruz Biotechnology, Dallas, TX, USA, 42‐T; 1 : 200) and anti‐HSP90 (Santa Cruz Biotechnology SC‐7947; 1 : 500). Detection of the chemiluminescent signal was performed with ChemiDoc Imaging System (Bio‐Rad, Hercules, CA, USA).

### 2.17 *In vitro* drug screenings

AGI‐24512 was purchased from MedChem Express (Monmouth Junction, NJ, USA, Cat. #: HY‐112130), while MRTX1719 was kindly provided by Mirati Therapeutics under an academic material transfer agreement. CRC cells were seeded at different densities (1.5–3 × 10^3^ cells/well) in 100 μL medium containing 10% FBS in 96‐multiwell plates at day 0. The following day, serial dilutions of the indicated drugs (AGI‐24512 or MRTX1719) in serum‐free medium were added to the cells (ratio 1 : 1) in technical triplicates, while DMSO‐only treated cells were included as controls. Cell viability was assessed after 7 days by measuring ATP content through Cell Titer‐Glo^®^ Luminescent Cell Viability assay (Promega) according to the manufacturer's protocol. Luminescence was measured by the Tecan SPARK M10 plate reader (Tecan, Männedorf, Switzerland). Cell viability measured for each treatment condition was normalized to viability of DMSO‐treated controls. Data represent average ± SD of at least three independent biological replicates. To compare the mean of the reduction of the cell viability between *MTAP*‐deleted and wild‐type CRC cell lines, for each inhibitor a one‐way ANOVA test has been performed at different drug concentrations with the formula Viability ~ Condition + Error (Model). This has required selecting few relevant doses among those we considered in our screening. In the case of the AGI‐24512 inhibitor, we have taken into account the ones closest to 1 μm since this concentration has been previously used in functional assays [46]. Instead, as for the MRTX1719 inhibitor, we have considered four concentrations (9.14, 27.43 , 82.3, and 246.9 nm) at which it is expected to be highly selective in inhibiting PRMT5 activity and cell viability in *MTAP*‐deleted cells, based on recently published results in HCT116 isogenic cell lines [47].

In addition, the response to temozolomide (TMZ) was retrieved from a previous publication in which 47 CRC cell lines were tested with TMZ in long‐term colony‐forming assays, from which half‐maximal inhibitory concentration (IC_50_) values were obtained [48].

### 2.18 Transcriptome‐wide gene expression outlier analysis in CRC samples

Gene expression quantification data (STAR counts) generated by The Cancer Genome Atlas (TCGA) project were downloaded for TCGA‐COAD and TCGA‐READ samples from Genomic Data Commons (GDC) using the genomicdatacommons Bioconductor package (Data Release 37). Data corresponding to primary tumors were selected, and those corresponding to patients for which RNA‐seq has been performed multiple times from the same or different vials were excluded. Then, data were processed as described above using GENCODE v36 as gene annotation to be consistent with the GDC mRNA quantification analysis pipeline. Finally, extreme positive and negative outliers were identified by adapting the previously described pipeline. In brief, after the Tukey's rule had been applied to the expression profile of each individual gene, the furthest outliers were selected for each gene and then further filtered based on differential and absolute expression values. In this case, only positive outliers whose differential expression level was higher than 2 were maintained, while negative outliers were kept if the absolute value of differential expression level was higher than 1. These alternative thresholds were chosen so that the percentage of extreme positive and negative outliers compared to the total number of positive and negative outliers in the TCGA dataset is similar to that observed in the CRC cell line dataset.

## 3 Results

### 3.1 Multi‐omics profiling of 226 CRC cell lines

**Fig. 1:**
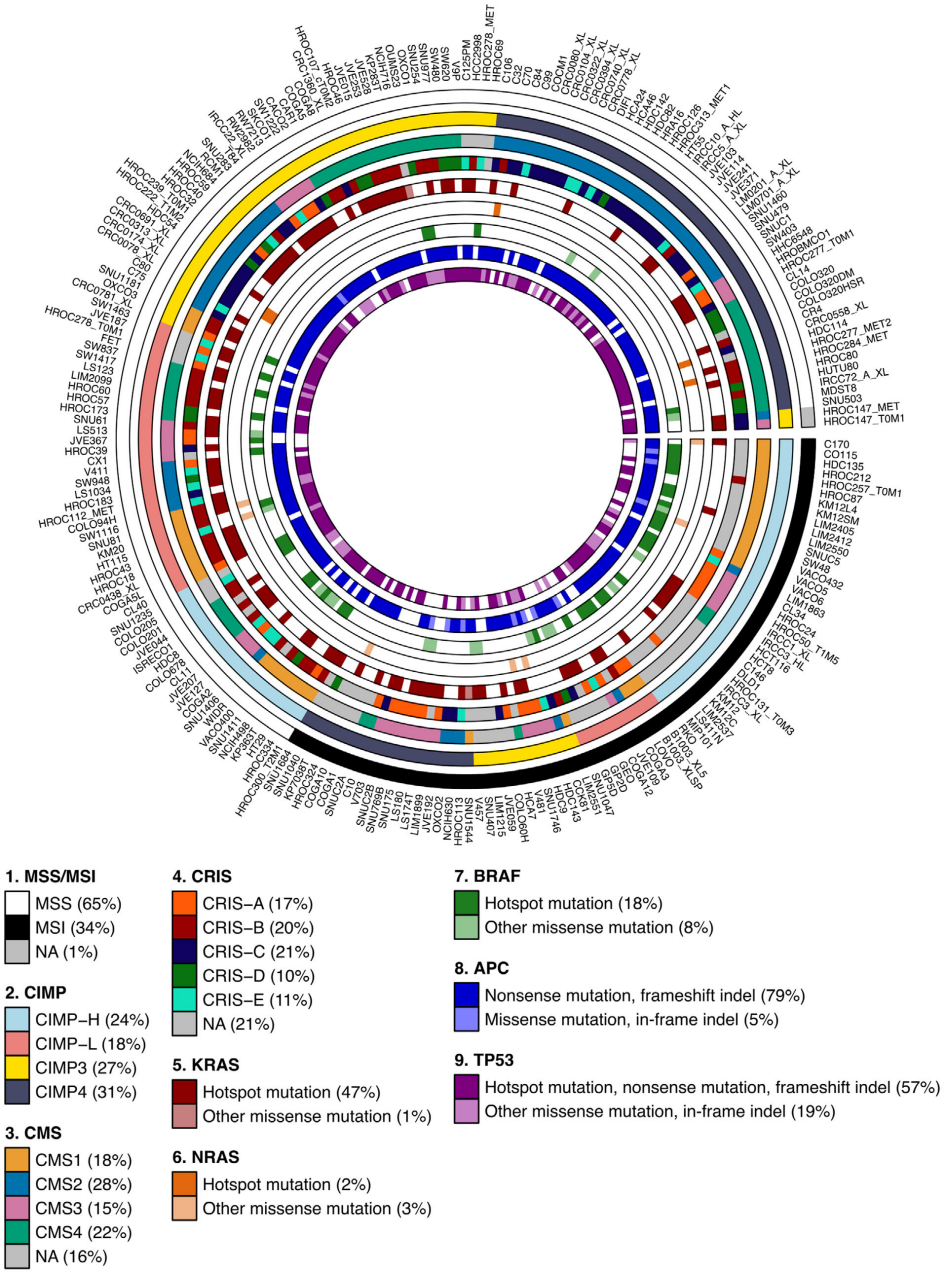
Genetic, transcriptomic, and epigenetic features of 226 colorectal cancer (CRC) cell lines. Circos plot delineating clinically relevant molecular features of 226 CRC cell lines. Each layer corresponds to a different genetic, transcriptomic, or epigenetic feature, organized from the outside to the inside in the following order: microsatellite status as microsatellite stable (MSS) or instable (MSI); *KRAS* mutational status; *NRAS* mutational status; *BRAF* mutational status; *APC* mutational status; *TP53* mutational status; consensus molecular subtypes (CMS) as CMS1, CMS2, CMS3, or CMS4; CRC intrinsic subtypes (CRIS) as CRIS‐A, CRIS‐B, CRIS‐C, CRIS‐D, or CRIS‐E; classification based on the CpG island methylator phenotype (CIMP) as CIMP‐H, CIMP‐L, CIMP3, or CIMP4. In the layers corresponding to CMS and CRIS gene expression‐based subtypes, CRC cell lines that cannot be confidently assigned to a single subtype (FDR > 5%) were labeled as NA (not available).

In this study, we analyzed 226 CRC cell lines collected from different sources or established in our laboratory starting from biopsy or surgical samples, as well as from patient‐derived xenografts (Fig. 1 and Table S1) [17, 19]. To check the genetic identity of the cell lines and to assess whether they are representative of CRC subtypes with clinical relevance, we performed STR profiling and assessment of the microsatellite status. STR profiling highlighted a small subset of genetically identical cell lines, including models previously reported as being established from the same patient (Table S2) [17]. In addition, we found that 148 (65%) models were MSS, while 76 (34%) displayed microsatellite instability (Fig. 1 and Table S1). Two models derived from the same patient (HROC147_T0M1 and HROC147_MET) displayed an intermediate profile, and their MSS or MSI status was not uniquely attributed.

Next, data obtained by WES, RNA‐seq, and DNA methylation microarray experiments were analyzed. Genetic alterations associated with CRC onset and progression were found at high frequency, including activating mutations in *KRAS*, *NRAS*, and *BRAF* genes, as well as mutations in *APC* and *TP53* genes that likely determine protein loss of function (Fig. 1 and Table S1). Furthermore, a gene expression‐based hierarchical clustering confirmed that CRC cell lines that originated from the same patient (as identified by the same STR profile) preferentially clustered together (Fig. S1) [17]. We next assessed whether gene expression‐based CRC subtypes (CMS and CRIS) found in tumor samples could be detected in preclinical models [32, 33, 49]. In total, 189 and 178 CRC cell lines were assigned to a specific CMS or CRIS class, respectively (Fig. 1 and Table S1). We also confirmed some known relationships between CMS and CRIS classifications, thus validating the analysis (Fig. S2). Finally, we took advantage of DNA methylation microarray data to classify CRC cell lines with respect to CIMP that is a distinguishing feature of the serrated pathway in colorectal tumorigenesis (Fig. 1, Fig. S3 and Table S1) [40].

### 3.2 Landscape of gene expression outliers in CRC cell lines

**Fig. 2:**
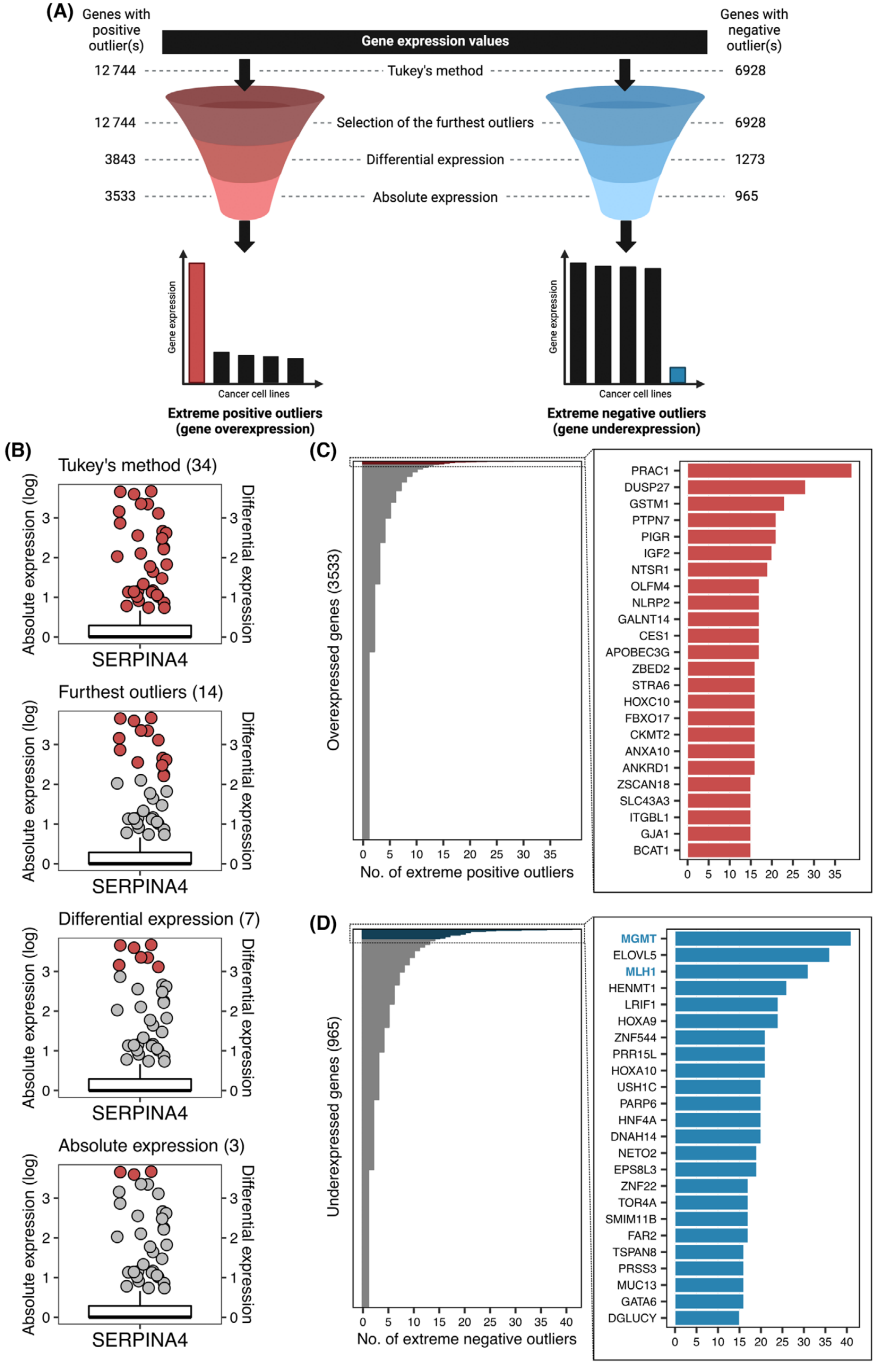
Identification of overexpression and underexpression events in 226 colorectal cancer (CRC) cell lines. (A) Graphical representation of the computational pipeline. The number of genes for which positive or negative outliers(s) were found after each step of the pipeline is shown on the left and the right sides, respectively. (B) Identification of extreme positive outliers for the *SERPINA4* gene. Each boxplot shows the distribution of *SERPINA4* expression values. Red dots are samples selected as positive outliers after each step of the pipeline. Their number is also reported between brackets in the title of each subpanel. (C) In the left subpanel, the number of extreme positive outliers is shown for each overexpressed gene. A detailed representation is reported for genes with the most elevated outlier frequency in the right subpanel (number of outlier samples ≥ 15). (D) In the left subpanel, the number of extreme negative outliers is shown for each underexpressed gene. A detailed representation is reported for genes with the most elevated outlier frequency in the right subpanel (number of outlier samples ≥ 15).

We have devised a computational workflow to systematically identify overexpression and underexpression events in cancer cells (Fig. 2A). Initially, transcriptome‐wide positive and negative gene expression outliers are mapped by applying the Tukey's rule to the expression profile of individual coding genes. We reasoned that this set of gene expression outliers may include functional events, such as alterations associated with oncogenic dependency or cancer cell‐specific vulnerabilities, alongside neutral events that reflect technical or biological noise. Therefore, a multi‐filter strategy is implemented to select extreme gene expression outliers, based on the hypothesis that they are more likely to be functionally relevant in cancer cells. First, when multiple samples are scored as positive or negative outliers for an individual gene, only the furthest outliers are kept. In detail, the positive outliers of any gene can be divided into two groups, those that are nearer to the maximum expression value and those that instead are nearer to the highest value that is not an outlier. The former are the furthest outliers of that gene. Similarly, the furthest negative outliers of any gene are those nearer to the minimum expression value than to the lowest value obtained after excluding outliers. Next, additional filters are performed taking into consideration both differential and absolute expression values (see Section 2.11).

When this strategy was applied to gene expression data from our collection of 226 CRC cell lines, positive and negative gene expression outliers were identified for 12 744 and 6928 genes, respectively, out of a total of 16 392 analyzed genes (Fig. 2A). This included 5001 genes for which both positive and negative gene expression outliers were found. After filtering, we retained 3533 and 965 genes that had extreme positive or negative gene expression outliers, respectively (Fig. 2A and Table S3). For example, the application of the Tukey's method resulted in the pinpointing of 34 CRC cell lines as positive outliers for the *SERPINA4* gene (Fig. 2B). The number of outliers progressively reduced at each filtering step and in the end three CRC cell lines were selected as “extreme positive outliers” for the *SERPINA4* gene (Fig. 2B). Similarly, two CRC cell lines were scored as “extreme negative outliers” for the *CNKSR1* gene starting from 10 samples that were considered as negative outliers based on the application of the Tukey's method (Fig. S4). Of note, only 138 genes had both extreme positive outliers and extreme negative outliers. We also observed that in most cases only one or two CRC cell lines were scored as extreme outliers for individual genes, but we found two subsets of genes for which extreme positive or negative outliers were identified at a relatively high frequency (Fig. 2C,D). Globally, every CRC cell line was scored as extreme positive outlier for at least one gene, and at least one underexpressed gene was found in virtually all samples (223 out of 226). The median number of overexpressed genes per cell model was 24, while the median number of underexpressed genes per cell line was 7.

We also characterized each CRC cell line by taking into account the corresponding amount of aberrantly expressed genes to compute positive, negative, or total outlier burden values. Finally, we looked for correlations with known molecular features of CRC cell lines (Fig. S5A–I). While the total outlier burden is not affected by the MSS or MSI status of CRC cell lines (Fig. S5A), we observed that it significantly changes between CRC cell lines that belong to different transcriptional subtypes (Fig. S5B,C). In particular, we found that the positive outlier burden is significantly higher in CMS4 CRC cell lines than in samples classified in other CMS subtypes (Fig. S5D). Consistently with that, when the CRIS classification is considered, the CRIS‐D subtype is the one with the highest positive outlier burden values (Fig. S5E). CMS4 CRC cell lines are also characterized by a significantly higher fraction of overexpressed genes whose overexpression only occurs in CMS4 samples (Fig. S5F). This suggests that the larger number of transcriptional alterations found in CMS4 samples reflects the activation of biological processes that are distinctive of this subtype. When we restricted the analysis to extreme negative outliers, we found that the negative outlier burden changes based on the CIMP status of CRC cell lines (Fig. S5G). In particular, it is slightly higher in CIMP‐H samples than in samples belonging to other DNA methylation‐based classes (Fig. S5H).

### 3.3 Method validation based on the identification of gene expression abnormalities associated with clinically relevant features of CRC cell lines

**Fig. 3:**
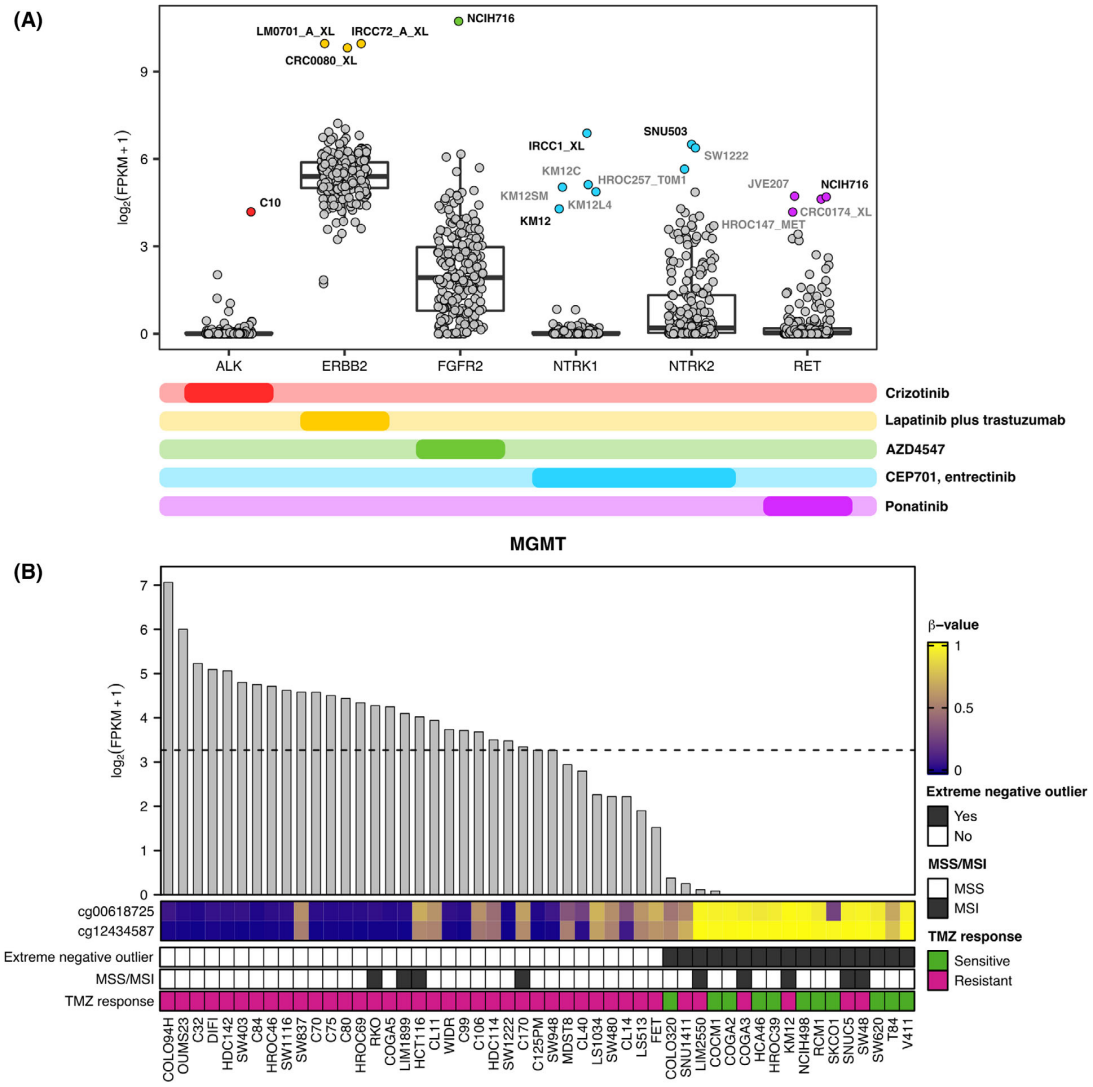
Gene expression abnormalities associated with clinically relevant features of colorectal cancer (CRC) cell lines. (A) Boxplots depicting the distribution of expression values of tyrosine kinase (TK) genes whose overexpression has been previously associated with oncogene addiction in CRC cells. For each TK gene, extreme positive outliers are highlighted with dots of a particular color, while other samples are reported as gray dots. The name of each CRC cell line selected as extreme positive outlier is also indicated (in gray those that were not included in a previous drug screening). The bottom panel summarizes the matching between overexpressed TK genes and kinase inhibitors whose effect on cell viability has been previously assessed. (B) Heatmap showing DNA methylation levels of promoter probes that were found differentially methylated between *MGMT* extreme negative outliers and other samples. DNA methylation levels were measured as β‐values and are represented using a color scale from dark blue (low DNA methylation level) to yellow (high DNA methylation level). *MGMT* expression profile is shown above the heatmap and sample ordering is the same in the two graphs, from high to low *MGMT* expression levels. Annotation bars below the heatmap indicate – from top to bottom – samples that were scored as *MGMT* extreme negative outliers, the microsatellite status of each sample, and sample response to temozolomide (TMZ). Only CRC cell lines that were previously tested for TMZ sensitivity are shown in the figure.

To validate our approach, we checked whether transcriptional alterations that are associated with known clinically relevant features of CRC cell lines are recapitulated by our atlas of extreme gene expression outliers. First, we considered a group of tyrosine kinase (TK) genes whose overexpression in CRC leads to oncogene addiction (*ALK*, *ERBB2*, *FGFR2*, *NTRK1*, *NTRK2*, and *RET*) [17, 19, 50, 51]. Importantly, the extreme positive outliers identified for these TK genes included cell models that we found to be highly sensitive to the corresponding kinase inhibitors (Fig. 3A) [17, 19, 50, 51]. In line with a previous result and consistently with the fact that they are genetically identical cell lines, *NTRK1* overexpression was found in all CRC cell lines of the KM12 family (KM12, KM12C, KM12SM, and KM12L4), although the effect of NTKR1 inhibition has been previously tested only in the KM12 CRC cell line (Fig. 3A) [17]. Similarly, few CRC cell lines in which we have not previously assessed the effect of kinase inhibitors were pinpointed as extreme positive outliers for the *NTRK2* and *RET* genes (Fig. 3A).

Regarding underexpression events, we noted *MLH1* and *MGMT*, whose status has a known predictive or prognostic role in CRC patients, in the subset of genes for which a relatively high number of CRC cell lines have been recognized as extreme negative outliers (Fig. 2C,D) [12, 52]. *MLH1* loss of expression was detected in 31 CRC cell lines and, as expected, microsatellite instability was observed in all *MLH1* extreme negative outliers (Fig. S6). On the other hand, 41 CRC cell lines were scored as *MGMT* extreme negative outliers. Of note, a subset of CRC cell lines labeled as *MGMT* extreme negative outliers was previously tested with TMZ: while MSS and MGMT‐deficient CRC cell lines are mostly sensitive to TMZ, the MSI status confers resistance to TMZ regardless of *MGMT* silencing (Fig. 3B) [48].

We then compared our workflow with an alternative method for detecting aberrantly expressed genes. Specifically, we selected OUTRIDER since it was developed to identify outlier samples specifically in the case of RNA‐seq expression data and we assessed its performance in identifying the molecular features that we just presented [42]. Overall, OUTRIDER found fewer outlier samples for fewer genes: specifically, 2049 overexpressed genes were found in 148 CRC cell lines, while 836 underexpressed genes were identified in 157 models. Importantly, OUTRIDER failed in identifying several of the clinically relevant and previously validated features of CRC cell lines that we mentioned above. In detail, OUTRIDER recognized as outliers for the *ERBB2* gene all the models with known *ERBB2* overexpression, but only three out of five models with *NTRK1* overexpression, and it did not pinpoint any of the models with *NTRK2*, *FGFR2*, or *RET* overexpression that are responsive to the pharmacological inhibition of the corresponding TK proteins (Fig. S7A–F). Moreover, according to the OUTRIDER output, *MLH1* and *MGMT* loss of expression does not occur in CRC cell lines (Fig. S7G,H).

In summary, the strategy that we have devised was able to correctly identify gene expression abnormalities that have been previously associated with clinically relevant features of CRC cell lines, thus supporting its effectiveness. In addition, it outperformed an alternative algorithm for gene expression outlier analysis.

### 3.4 Aberrant DNA methylation in promoter regions underlies outlier expression values that are recurrently found in CRC cell lines

**Fig. 4:**
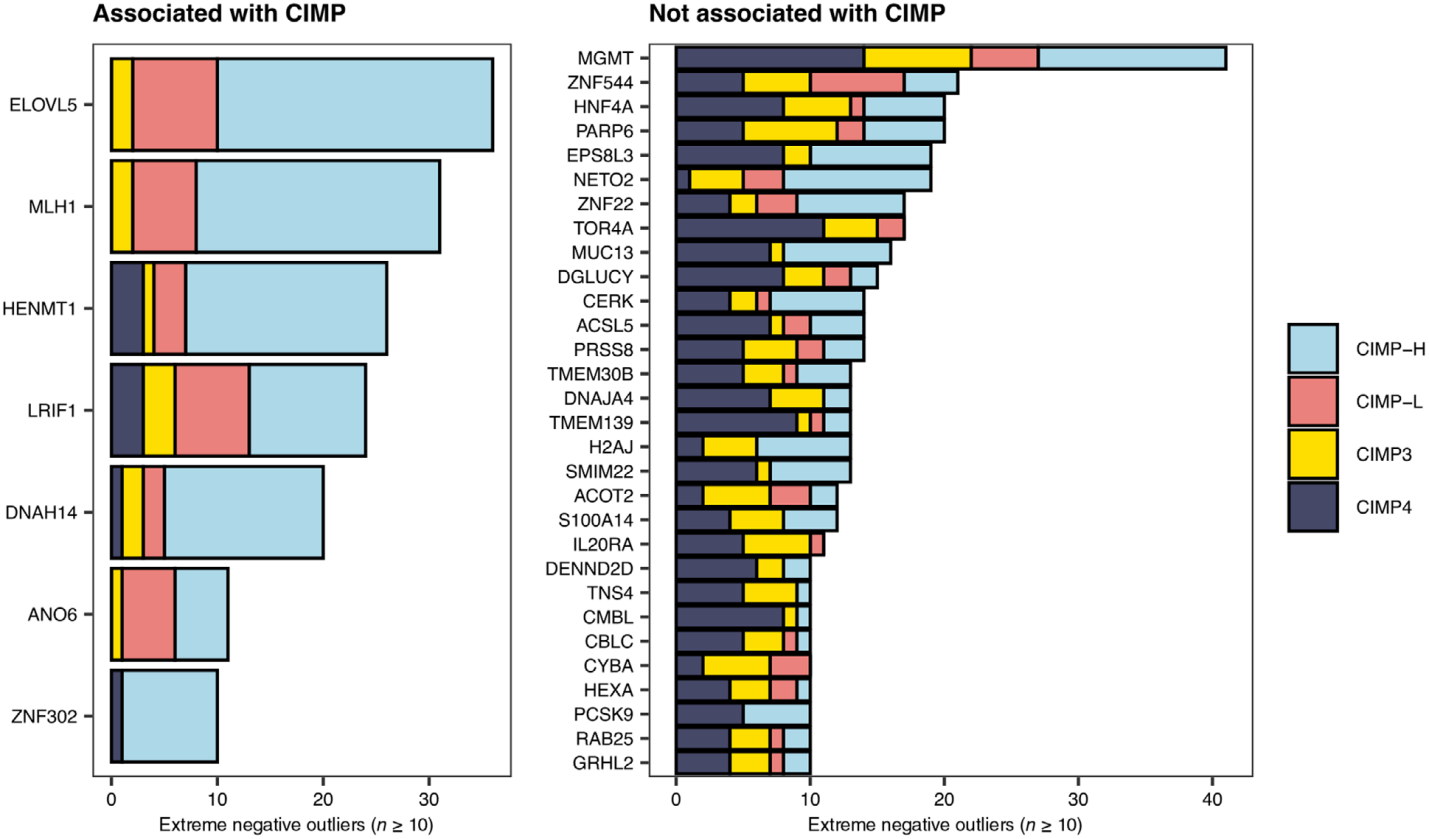
Promoter hypermethylation in extreme negative outliers occurs concomitantly with or independently from the CpG island methylator phenotype (CIMP). Genes whose underexpression events were associated with promoter hypermethylation were classified as CIMP‐associated (left subpanel) or not CIMP‐associated (right subpanel) based on the enrichment of CIMP‐positive samples (CIMP‐H and CIMP‐L) in extreme negative outliers. Each bar depicts the CIMP classes that were attributed to the extreme negative outliers of a single gene. Only genes for which at least 10 colorectal cancer (CRC) cell lines were identified as extreme negative outliers are shown in the figure.

Epigenetic modifications critically control gene expression levels [53]. Therefore, we assessed whether extreme gene expression outliers are characterized by aberrant DNA methylation in promoter regions of overexpressed or underexpressed genes. First, DNA methylation microarray data analysis confirmed high DNA methylation levels in *MLH1* or *MGMT* promoter regions in CRC cell lines recognized as extreme negative outliers for each of these genes (Fig. 3B and Fig. S6). Then, the analysis was extended to all genes for which at least two CRC cell lines were pinpointed as extreme positive or negative outliers, thus including both overexpression and underexpression events. Globally, this revealed 198 genes for which underexpression in extreme negative outliers was associated with high DNA methylation levels in promoter regions (promoter hypermethylation) (Table S4). For example, few CRC cell lines (HROC278_MET, HROC278_T0M1, HROC334, and KP363T) were selected as extreme negative outliers for the *RAD51C* gene. In previous works, we found that these cells are highly sensitive to inhibition of PARP and other proteins involved in the DNA damage response [54, 55]. Here, we observed that in these models *RAD51C* silencing is associated with promoter hypermethylation, a feature previously detected in cancer types other than CRC (Fig. S8) [14]. In addition, we found 102 genes for which the reduction of DNA methylation levels in promoter regions (promoter hypomethylation) likely contributed to overexpression in extreme positive outliers (Table S4). We also examined the position of each individual CpG within the promoter regions by taking into account CpG islands, shores, shelfs, and open sea. In the case of both extreme positive outliers and extreme negative outliers, we observed that the distribution in the different regions differs between CpG sites that are or not differentially methylated between outlier and other samples (chi‐square *P*‐value = 0.00023 and 7.6e‐12, respectively). In general, in both cases, the majority of differentially methylated CpG sites are located within CpG islands (Fig. S9 and Table S4). In addition, we found that CIMP‐H samples are characterized by a slightly higher fraction of underexpressed genes whose silencing is associated with promoter hypermethylation (Fig. S5I). This is consistent with the global genome hypermethylation that characterizes this subset of CRC samples [40]. Finally, we considered the extreme negative outliers of genes for which we found a correspondence between underexpression and promoter hypermethylation and tested whether they were significantly enriched for CIMP‐positive samples (CIMP‐H and CIMP‐L). Using this strategy, we were able to distinguish 23 genes associated with CIMP and 138 genes that were silenced through promoter hypermethylation in CRC cell lines independently from CIMP (Fig. 4 and Table S5).

### 3.5 Outlier expression values are associated with genomic rearrangements

Large genetic abnormalities, such as gene amplifications, gene deletions, and gene fusions, are a potential source of gene expression outliers [17, 19, 56]. Therefore, we assessed their impact in our collection of CRC extreme expression outliers (Table S3).

**Fig. 5:**
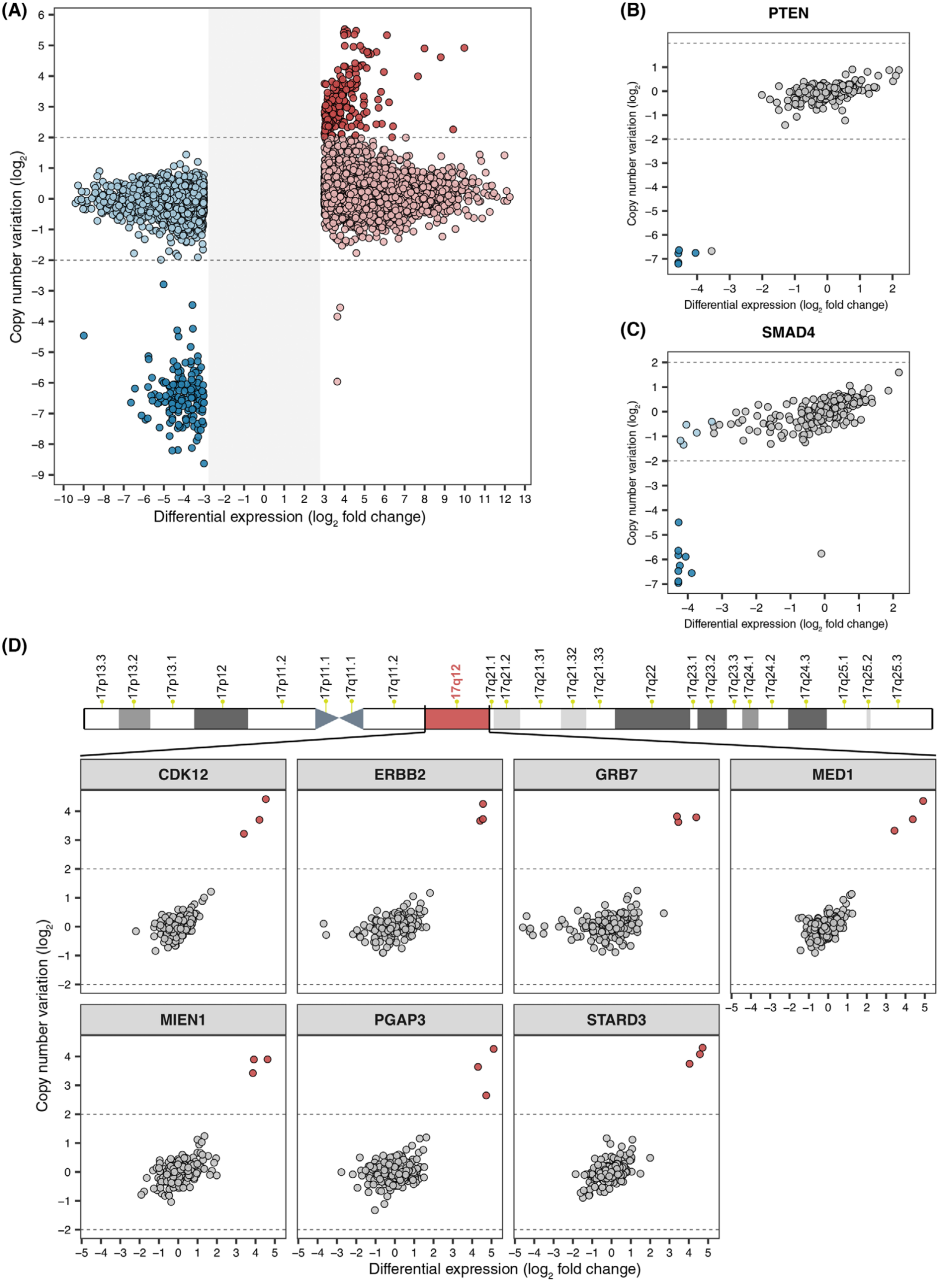
Gene amplifications and deletions leading to overexpression and underexpression events. (A) Scatter plot of all the extreme positive (red) and negative (blue) gene expression outliers that were identified. The outliers of each gene are defined by differential expression values (*x*‐axis), measured as log_2_ fold change with respect to the median gene expression, and log_2_‐transformed copy number variation (CNV) values (*y*‐axis). The gray area in the middle corresponds to the interval where extreme positive and negative outliers cannot be located according to the filters that were applied. Horizontal dashed lines correspond to thresholds used in calling gene amplifications and deletions. Outliers in which either gene overexpression was associated with gene amplification or gene underexpression was associated with gene deletion are shown in darker colors. (B) Scatter plot of *PTEN* differential expression values, measured as log_2_ fold change with respect to the median expression, and CNV values in 226 colorectal cancer (CRC) cell lines. Samples in which both *PTEN* underexpression and *PTEN* genetic deletion were found were colored in dark blue. (C) Scatter plot of *SMAD4* differential expression values, measured as log_2_ fold change with respect to the median expression, and CNV values in 226 CRC cell lines. *SMAD4* extreme negative outliers are blue colored and a darker color is used for samples in which both *SMAD4* underexpression and *SMAD4* genetic deletion were found. (D) Scatter plots of differential expression values, defined as log_2_ fold change with respect to the median gene expression, and log_2_‐transformed CNV values in 226 CRC cell lines for genes that are co‐overexpressed and co‐amplified with *ERBB2* due to genome proximity. Extreme positive outliers are red colored. The ideogram of chromosome 17 with G‐banding pattern is shown at the top and the genomic region in which the selected genes are located (chromosome 17q12) is highlighted in red.

First, we identified 174 genes whose overexpression was associated with gene amplification in at least some extreme positive outliers and 104 genes whose underexpression was concomitant with genetic deletion (Fig. 5A). In line with the concept that tumor suppressor genes are frequently lost or inactivated during colorectal tumorigenesis, *PTEN* and *SMAD4* were among the recurrently underexpressed and deleted genes in CRC cell lines [57, 58]. In detail, five CRC cell lines were scored as *PTEN* extreme negative outliers and exhibited genetic deletion at the genome locus that includes *PTEN* (HROC277_MET2, KP283T, OUMS23, SNU479, and SW1222) (Fig. 5B). Additionally, consistent with the fact that the HROC277_T0M1 and HROC277_MET2 CRC cell lines were derived, respectively, from the primary tumor and a metastatic lesion obtained from the same patient, *PTEN* genetic deletion was also found in the HROC277_T0M1 model that had very low *PTEN* expression. A total of 14 CRC cell lines were scored as *SMAD4* extreme negative outliers and 9 of them were also characterized by *SMAD4* genetic deletion (COLO678, HROC239_T0M1, HROC284_MET, HROC300_T2M1, HROC59, IRCC10_A_HL, KP363T, SNU1181, and SNU1411) (Fig. 5C). In addition, our analysis revealed several co‐occurring overexpression or underexpression events due, respectively, to co‐amplification or co‐deletion of genes that are located in the same genomic regions. For example, three CRC cell lines were found as *ERBB2* extreme positive outliers (CRC0080_XL, IRCC72_A_XL, LM0701_A_XL), and all of them were also characterized by overexpression and amplification of six additional genes (*CDK12*, *GRB7*, *MED1*, *MIEN1*, *PGAP3*, and *STARD3*) that are positioned in the same genomic region as the *ERBB2* gene (chromosome 17q12) (Fig. 5D).

**Fig. 6:**
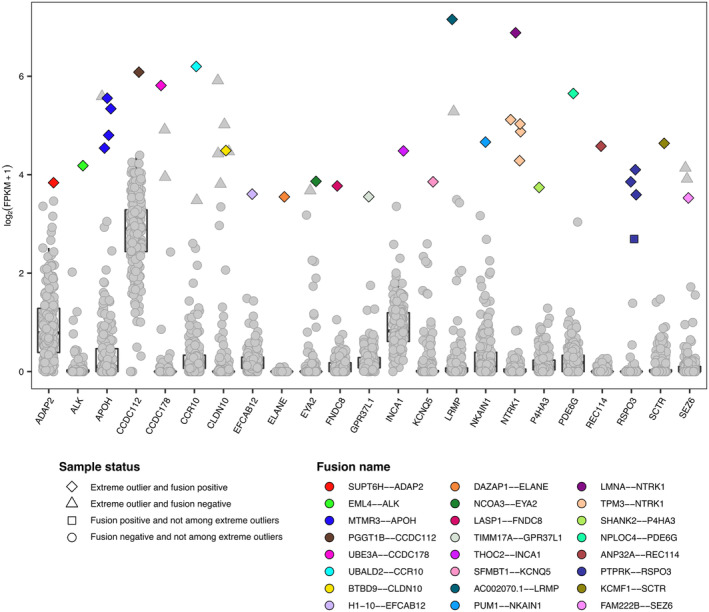
Somatic fusion transcripts associated with the overexpression of 3′ partner genes in extreme positive outliers. Boxplots depicting the distribution of expression values for genes whose overexpression was associated with a somatic fusion transcript in extreme positive outliers. Different point shapes are used to distinguish colorectal cancer (CRC) cell lines based on their gene‐specific status. In addition, fusion‐positive extreme positive outliers are shown in different colors according to the somatic fusion transcript that was identified.

Next, RNA‐seq data were leveraged to identify somatic fusion transcripts in CRC cell lines, and we checked whether the presence of each fusion transcript matched with the overexpression of the 3′ partner gene. It is indeed possible that its expression comes under the control of a stronger promoter if the fusion transcript results from a genomic rearrangement. We found 24 gene fusions associated with the overexpression of 23 genes in 30 CRC cell lines (Fig. 6). We observed that the majority of them involve 5′ partner a gene that is normally expressed in CRC cells and 3′ partner a gene that is not expressed in the majority of models (median FPKM < 1) (Fig. S10). We also confirmed the presence of actionable gene fusions that we functionally characterized in previous studies, namely *EML4‐ALK*, *LMNA‐NTRK1*, and *TMP3‐NTRK1* [17, 50]. In addition, the *PTPRK3*‐*RSPO3* gene rearrangement was found in two previously characterized models (SNU1411 and CRC0781_XL) [19, 59] and also found in the HROC300_T2M1 cell line. Finally, the *MTMR3‐APOH* fusion was detected in four CRC cell lines (CX1, HT29, KM20, and WIDR) that were found as *APOH* extreme positive outliers, thus further validating the approach as these cell lines were derived from the same patient.

### 3.6 Analysis of overexpressed enzyme genes pinpoints therapeutic targets

**Fig. 7:**
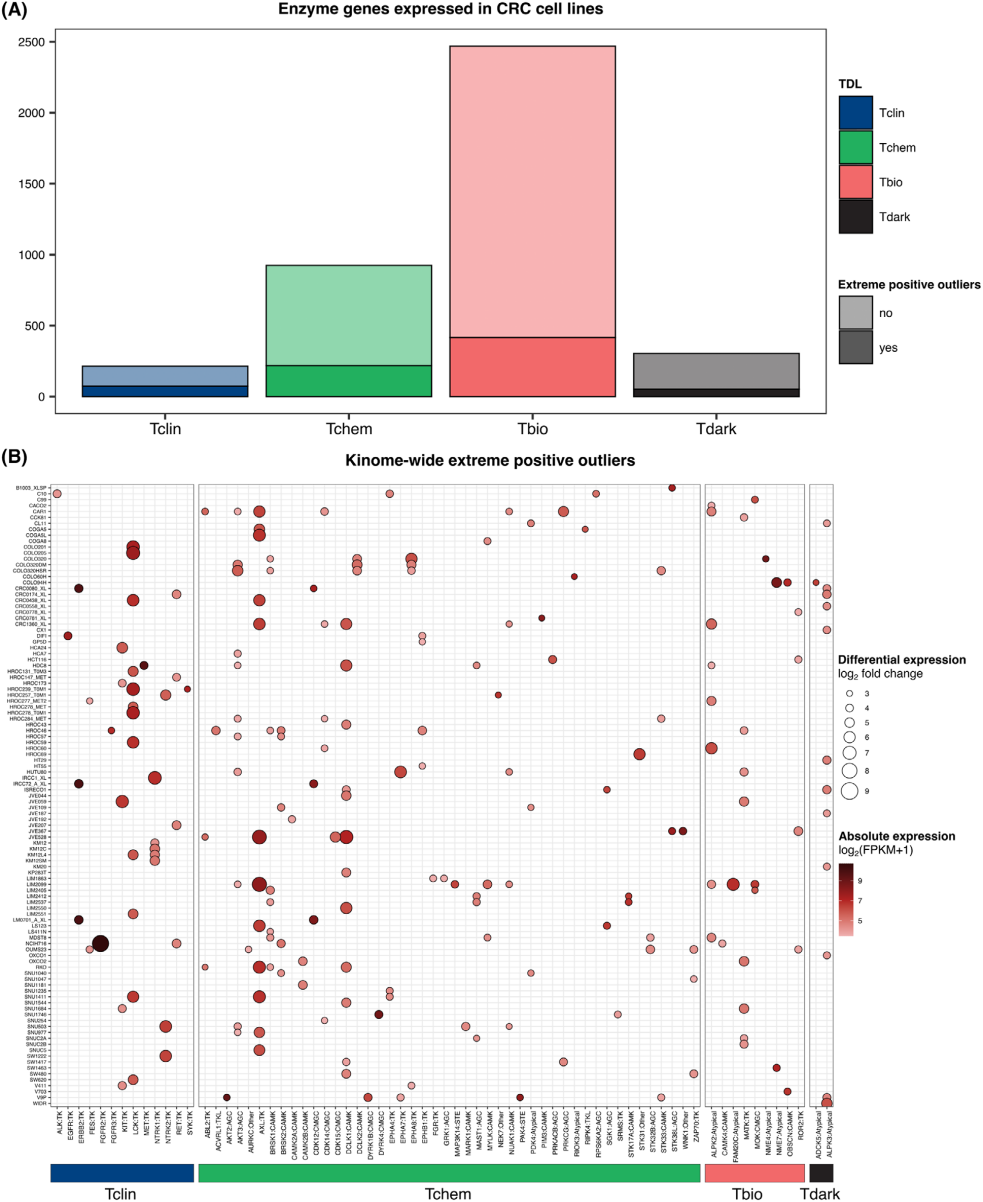
Target developmental level (TDL) categories applied to overexpressed enzyme genes. (A) Enzyme genes expressed in our colorectal cancer (CRC) cell line collection were categorized into four classes according to their TDL, from high (Tclin) to low (Tdark) depth of clinical, biological, and chemical investigation. Each bar corresponds to the number of expressed enzyme genes for each TDL class and the fraction of genes for which extreme positive outliers were found is highlighted with darker colors. (B) Bubble chart of extreme positive outliers identified for kinase genes (kinome‐wide extreme positive outliers). The outliers of each kinase gene are defined by absolute gene expression (FPKM value) and differential expression (log_2_ fold change with respect to the median gene expression). Overexpressed kinase genes are categorized into the four TDL classes and reported along with the name of the kinase families.

As discussed above, the identification of overexpressed genes may unveil new drug targets. Here, we focused on genes predicted to encode proteins endowed with enzymatic activity, since they are ideally suited for pharmacological inhibition. As a first step, we applied the classification scheme proposed by the Illuminating the Druggable Genome (IDG) initiative for target prioritization [44]. In brief, human proteins have been organized into four categories (Tclin, Tchem, Tbio, and Tdark) based on the TDL that measures the depth of clinical, chemical, and biological investigation associated with each protein. Starting from 20 375 human proteins extracted from the Swiss‐Prot UniProtKB database, we found 3704 enzyme genes expressed in CRC cell lines, and we identified at least one overexpression event for 759 enzyme genes (Table S3). Overexpressed enzyme genes were distributed in all TDL categories, including enzymes for which clinically approved drugs or small molecules are already available: 73 and 218 genes indeed belonged to the Tclin and Tchem categories, respectively (Fig. 7A and Table S3). Overexpressed kinase genes were also found across all TDL categories, but the TK family is the only one found in the Tclin category that, as expected, included the clinically relevant kinases that we discussed above (Fig. 7B and Table S3). We also found cell models bearing overexpression of additional TK genes that are included in the Tclin class (*FES*, *FGFR3*, *LCK*, *MET*, and *SYK*) and other kinases for which small molecules are available (Tchem category) (Fig. 7B and Table S3).

### 3.7 Characterization of underexpressed genes unveils synthetic lethal vulnerabilities in CRC cells

**Fig. 8:**
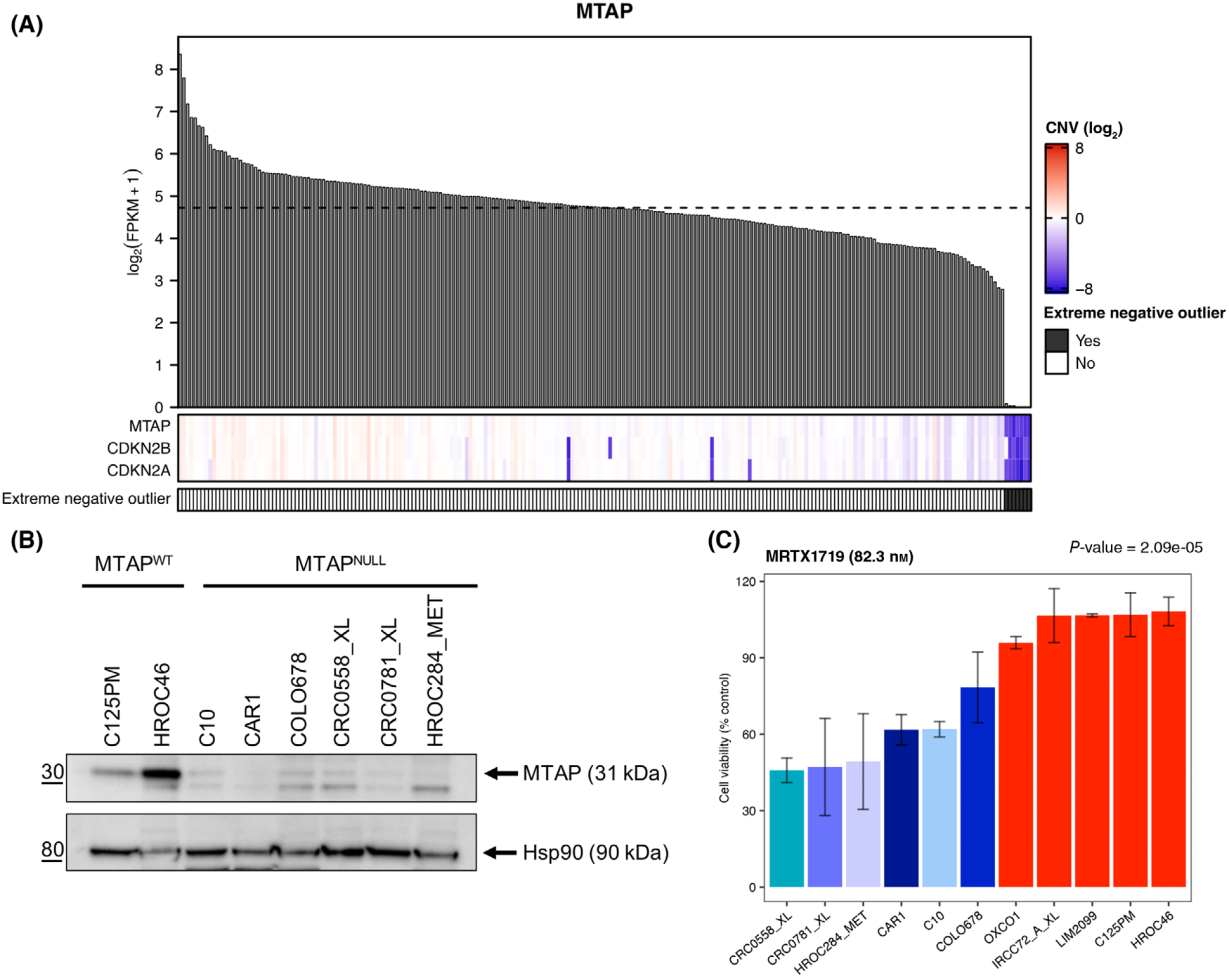
*MTAP*‐deleted colorectal cancer (CRC) cell lines are sensitive to the MRTX1719, an inhibitor of the PRMT5:MTA complex. (A) Heatmap showing log_2_‐transformed copy number variation (CNV) values of *MTAP*, *CDKN2A*, and *CDKN2B* genes in 226 CRC cell lines. Log_2_‐transformed CNV values are represented using a color scale from red (gene amplification) to blue (gene deletion), and white indicates copy number neutrality. *MTAP* expression profile is shown above the heatmap, and sample ordering is the same in the two graphs, from high to low *MTAP* expression levels. The annotation bars below the heatmap indicate samples that were identified as *MTAP* extreme negative outliers. (B) MTAP protein expression in *MTAP*‐deleted CRC cell lines (MTAP^NULL^) and models in which the *MTAP* gene is normally expressed (MTAP^WT^). Hsp90 was used as loading control. (C) Cell viability (% control cells treated with DMSO) measured in *MTAP*‐deleted (blue bars) and wild‐type (red bars) CRC cell lines after 7‐day treatment with MRTX1719 at a relevant concentration (82.3 nm). Data represent mean ± SD of at least three independent biological replicates. The *P*‐value obtained with one‐way ANOVA test is reported.

Several studies have previously reported synthetic lethal interactions between metabolic enzymes [60, 61]. Intriguingly, we identified 274 enzyme genes that are underexpressed in one or multiple CRC cell lines (extreme negative outliers). In particular, seven CRC cell lines (C10, CAR1, COLO678, CRC0558_XL, CRC0781_XL, HROC284_MET, and RW2982) were identified as extreme negative outliers for the methylthioadenosine phosphorylase (*MTAP)* gene that codes for a key enzyme in the methionine salvage pathway (Fig. 8A). In the human genome, the *MTAP* gene is located at chromosome 9p21.3 adjacent to two tumor suppressor genes (*CDKN2A* and *CDKN2B*), and co‐deletion is frequently observed in cancer cells [62, 63]. Recent studies also reported that the dysregulated metabolic state of MTAP‐deficient cancer cells gives rise to a targetable vulnerability [62, 63]. The viability of *MTAP*‐deficient cells is indeed impaired by inhibition of the protein arginine methyltransferase 5 (PRMT5) enzyme whose activity can be reduced both indirectly and directly, through MAT2A inhibition or by targeting the PRMT5‐MTA complex that selectively appears in the absence of MTAP [46, 47, 64, 65]. First, we found that *MTAP*, *CDKN2A*, and *CDKN2B* were co‐deleted in *MTAP* extreme negative outliers (Fig. 8A), with *CDKN2A‐* and *CDKN2B*‐deleted CRC cell lines exhibiting very low or zero expression values (Fig. S11). Next, 6 *MTAP*‐deleted CRC cell lines were selected for biological characterization, along with five CRC cell lines (HROC46, C125PM, IRCC72_A_XL, LIM2099, and OXCO1) in which the *MTAP* gene is normally expressed. Since the *MTAP*‐deleted cell line RW2982 grows very poorly, it was excluded from further experiments. Loss of MTAP protein expression in *MTAP*‐deleted cell lines was confirmed by western blot (Fig. 8B). Then, we assessed whether inhibitors that determine reduction of PRTM5 activity are selectively active in *MTAP*‐deleted CRC cell lines. When the cell models were treated with increasing concentrations of an inhibitor of the metabolic enzyme MAT2A (AGI‐24512), we observed a modest growth reduction in five out of six *MTAP*‐deleted CRC cell lines, while viability of control cell lines was not affected (Fig. S12A). In line with what can be observed in the drug response curves, the difference between *MTAP*‐deleted and wild‐type CRC cell lines was statistically significant when two relevant concentrations were considered (Fig. S12B,C). We then treated the same subset of CRC cell lines with an inhibitor of the PRMT5:MTA complex (MRTX1719), a recently developed drug that has shown promising results in solid tumors [47, 65]. Notably, treatment with MRTX1719 was much more effective in limiting the growth of *MTAP*‐deleted CRC cell lines as compared with wild‐type cells (Fig. S12D). The difference was statistically significant at multiple drug concentrations (Fig. 8C and Fig. S12E–G).

### 3.8 Aberrantly expressed genes in both CRC cell lines and tissue samples obtained from colorectal tumors

To further assess the clinical relevance of our findings in CRC preclinical models, we checked whether extreme positive and negative outliers are found in human tumor samples for the same genes that are aberrantly expressed in cell lines. To this end, we obtained gene expression data of 611 primary colorectal tumors from The Cancer Genome Atlas (TCGA) project [57]. Positive and negative outliers were found for 14 913 and 7890 genes, respectively, when the Tukey's rule was applied to the expression profile of each individual gene. When we selected the furthest outliers for each gene, we observed that the deviation of positive and negative outliers from the other samples is usually less prominent in the TCGA dataset than in the CRC cell line dataset (Wilcoxon test *P*‐value < 2.2e‐308 in both cases) (Fig. S13). Therefore, we chose alternative thresholds to pinpoint the extreme outliers in the TCGA dataset, making sure that the fraction of extreme positive or negative outliers compared to the total number of positive and negative outliers was comparable to that obtained in the CRC cell line dataset. In this way, we identified 6005 and 3083 genes for which CRC samples were recognized as extreme positive or negative outliers, respectively. Importantly, in the TCGA dataset, we found extreme positive outliers for 2005 genes whose overexpression was also observed in CRC cell lines (56.75%), while we confirmed that the underexpression of 437 genes for which extreme negative outliers were found in the CRC cell line dataset also occur in human tumors (45.28%).

## 4 Discussion

Several molecular alterations that have been associated with clinically relevant features of human tumors lead to gene expression alterations, such as *ERBB2* amplification and *MGMT* promoter hypermethylation [7, 8, 13]. Furthermore, cancer cells can become highly dependent on dysregulated transcriptional programs independently from the genetic alterations that have induced the gene expression changes [15]. Here, we propose a novel computational workflow to identify and prioritize transcriptome‐wide gene expression outliers as a way to rapidly find genes whose alterations could give rise to therapeutic vulnerabilities in cancer cells. Importantly, extending the analysis beyond small groups of druggable genes such as kinases and searching both gene overexpression (positive outliers) and gene underexpression (negative outliers) events are crucial improvements with respect to previous studies [17, 18, 19, 20]. The approach can be exploited in different tumor types starting only from gene expression data. In this study, we decided to focus on CRC for which precision oncology has lagged behind with respect to other solid tumors [23].

Over the last years, we have assembled and annotated a panel of 226 CRC cell lines that well recapitulate the heterogeneity and molecular characteristics that are observed in CRC patients [17, 19]. The MSI prevalence is usually lower in CRC patients (5–15% depending on the disease stage) [57] as compared to our collection of CRC cell lines (34%). This discrepancy is most likely related to the higher capacity of MSI cell lines to adapt to *in vitro* growth conditions probably due to their higher genetic instability [66]. Notably, for each CRC cell line, we have obtained multi‐omics data that provides an integrated genetic, epigenetic, and transcriptional characterization which was not available for such a large dataset of CRC models.

When RNA‐seq expression data were analyzed, we found 3533 and 965 genes with extreme positive and negative gene expression outliers, respectively. Importantly, this analysis correctly identified several gene expression abnormalities of CRC cell lines that have been previously associated with clinically relevant features. This observation supports the effectiveness of the novel computational approach we developed. On the contrary, an alternative method for recognizing expression outliers in RNA‐seq data (OUTRIDER) failed in detecting many of them.

We then exploited the multi‐omics data to search for genetic and epigenetic alterations associated with extreme expression values. Specifically, we have considered alterations of DNA methylation levels at promoter regions, gene amplifications, gene deletions, and the presence of fusion transcripts. Different genomic rearrangement‐independent mechanisms were reported to describe the origin of fusion transcripts [67]. However, given the gene expression profiles and their position in the genome, it is plausible that genomic translocations account for the majority of the fusion transcripts that we identified. This is also in line with a previous pan‐cancer study [68]. Overall, about 6% and 3% of genes for which extreme positive outliers were found were connected with a genetic or epigenetic alteration, respectively. In addition, about 11% of genes for which underexpression events were identified were linked to a genetic alteration and about 21% of them were instead associated with an epigenetic alteration. It follows that neither genetic alterations nor epigenetic abnormalities were identified to explain the overexpression or the underexpression of 92% and 69% of genes for which extreme positive or negative outliers were, respectively, found. Some technical aspects may have limited our capability of identifying such events. For example, single‐end RNA‐seq data are suboptimal for the identification of fusion transcripts [34, 35]. Moreover, further associations between additional epigenetic features of CRC cell lines and outlier expression values could be identified if data other than DNA methylation would be available [69]. Nonetheless, our results prove that looking for transcriptome‐wide gene expression outliers is an effective strategy to discover features of cancer cells that may have a different origin. They also indicate that many extreme expression values of unknown functional relevance may not be easily predicted without directly parsing gene expression data.

We acknowledge that the approach that we propose has some limitations. For example, although numerous studies have shown that protein levels are primarily determined by transcript concentrations in steady‐state conditions [70], the translation of extremely high or low gene expression levels into abnormal protein abundance cannot be given for granted due to the complexity of gene expression regulation and the influence of other biological processes. Of interest, as demonstrated by several studies, the “extreme” expression outliers should in most instances lead to abnormal protein expression [17, 19, 48, 51].

By mining our atlas of CRC expression outliers, novel findings have emerged. First, by annotating overexpressed enzyme genes with the corresponding Target Development Level (TDL) [44], we pinpointed several proteins whose activity is expected to be dysregulated in CRC cells and for which drugs or active ligands are already available. On the other hand, the exploration of underexpression events may suggest novel synthetic lethal targets. As a proof of concept, we showed that two drugs (AGI‐24512 and MRTX1719) that inhibit PRMT5 activity selectively impaired cell viability of CRC cell lines recognized as extreme negative outliers for the *MTAP* gene that is co‐deleted with the *CDKN2A*/*CDKN2B* tumor suppressor genes. The most effective results were obtained with MRTX1719, which is currently being tested in a phase 1/2 clinical trial in patients with advanced solid tumors with homozygous genetic deletion of the *MTAP* gene [47, 65]. In our CRC cell line collection, the prevalence of *MTAP* loss (3%) is in line with that seen in mCRC patients for other molecular alterations that are targeted by clinically relevant treatments [23].

Many studies have demonstrated that cancer cell lines recapitulate the molecular features observed in human tumors, including gene expression and pharmacological profiles [17, 19, 71, 72]. However, we cannot exclude that some extreme gene expression outliers are artifacts due to adaptation to *in vitro* growth conditions. Therefore, we have verified how many expression abnormalities that we identified in CRC cell lines also occur in colorectal tumors by taking advantage of expression data from 611 TCGA samples. This required adapting the thresholds used in the selection of the extreme outliers to deal with the observation that the deviation of the furthest positive or negative outliers of each gene from the other samples is usually less prominent in the TCGA dataset than in the CRC cell line dataset. This likely occurs because gene expression values obtained through bulk RNA‐seq only reflect the average of signals from multiple cell types when tumor tissue specimens are analyzed. It should also be noted that two databases encompass samples derived from different stages of CRC progression: CRC cell lines were derived from both primary and metastatic tumors, while only primary tumors were analyzed in the TCGA dataset. We report that about half of the genes for which extreme positive or negative outliers were found in CRC cell lines are also overexpressed or underexpressed in human tumors, thus providing another validation of the clinical relevance of our atlas of CRC gene expression outliers.

## 5 Conclusions

We have devised a novel computational workflow in which a transcriptome‐wide analysis is performed to find extreme gene expression outliers displaying the overexpression or the underexpression of single genes. Subsequent characterization of dysregulated genes can be used to guide the discovery of novel drug targets and biomarkers in cancer cells. Specifically, in this study we present an atlas of CRC extreme expression outliers whose further exploration may lead to the identification of new actionable targets. The approach can be also exploited to prioritize candidates for drug development in other tumor types.

## Conflict of interest

AB served in a consulting/advisory role for Inivata and Guardant Health. AB is a member of the scientific advisory board of Neophore, Inivata, and Roche Genentech CRC Advisory Board. AB receipts grants/research supports from Neophore, Astrazeneca, and Boehringer. AB is cofounder and shareholder of NeoPhore Limited and shareholder of Kither. FDN received speaker's fees from Illumina and served in a consulting/advisory role for Amgen and Pierre Fabre Pharma. SA reports personal fees from MSD Italia and a patent (international PCT patent application No. WO 2023/199255 and Italian patent application No. 102022000007535) outside the submitted work. The remaining authors declare that they have no competing interests.

## Author contributions

AB, EM, FDN, and MR conceived and designed the study and interpreted the data. AB and EM wrote the manuscript with input from all authors. AL, CC, LB, and GGio performed experiments for multi‐omics characterization of the CRC cell line collection. GGra, MM, KB, NMR, PPV, and SA performed biological experiments and interpreted the data. EM, GGra, PA, GC, GR, and GCo performed bioinformatic analyses. EM, GGra, PA, and GC interpreted the results of bioinformatic analyses. ML provided CRC cell lines. All authors read and approved the final manuscript.

## Supporting information


**Fig. S1.** Gene expression‐based hierarchical clustering of 226 colorectal cancer (CRC) cell lines.
**Fig. S2.** Correspondences between consensus molecular subtypes (CMS) and CRC intrinsic subtypes (CRIS) identified in 226 colorectal cancer (CRC) cell lines.
**Fig. S3.** AnnotaGon of the CpG island methylator phenotype (CIMP) in 226 colorectal cancer (CRC) cell lines.
**Fig. S4.** Identification of extreme negative outliers for the CNKSR1 gene.
**Fig. S5.** Outlier burden values are associated with molecular features of colorectal cancer (CRC) cell lines.
**Fig. S6.** Promoter hypermethylation and microsatellite instability in MLH1 extreme negative outliers.
**Fig. S7.** Expression rank plots generated by Outlier in RNA‐seq Finder (OUTRIDER) for a selection of relevant genes.
**Fig. S8.** Promoter hypermethylation in RAD51C extreme negative outliers.
**Fig. S9.** Genomic position of analyzed CpG sites in transcript promoters with respect to CpG islands.
**Fig. S10.** Expression levels of genes involved in somatic fusion transcripts associated with gene overexpression in extreme positive outliers.
**Fig. S11.** CDKN2A and CDKN2B deletion is associated with very low or zero expression values in colorectal cancer (CRC) cell lines.
**Fig. S12.** AGI‐24512 and MRTX1719 screening in *MTAP*‐deleted and wild‐type colorectal cancer (CRC) cell lines.
**Fig. S13.** Positive and negative outliers found in The Cancer Genome Atlas (TCGA) dataset are usually less prominent than those found in the colorectal cancer (CRC) cell line dataset.
**Table S1.** Annotation of the 226 colorectal cancer (CRC) cell lines.
**Table S2.** Short tandem repeats (STR) profiles of the 226 colorectal cancer (CRC) cell lines considering 16 different loci.
**Table S3.** Atlas of colorectal cancer (CRC) extreme gene expression outliers.
**Table S4.** Differential methylation analysis for genes for which at least two colorectal cancer (CRC) cell lines were found as extreme positive (first sheet) or negative (second sheet) outliers.
**Table S5.** Enrichment of samples that are positive for the CpG island methylator phenotype (CIMP) among the extreme negative outliers of a single gene.

## Data Availability

Raw DNA methylation data (IDAT files) previously obtained for CRC cell lines using the Infinium HumanMethylation450 BeadChip array are available in the Gene Expression Omnibus (GEO) with GSE86078 accession code. Raw DNA methylation data (IDAT files) recently obtained for CRC cell lines using the Infinium MethylationEPIC BeadChip microarray were deposited on GEO with GSE220197 accession code. WES and RNA‐seq data previously obtained for CRC cell lines are available in the European Nucleotide Archive (ENA) with PRJEB33045 and PRJEB33640 accession codes. Further NGS data for CRC cell lines were deposited in ENA with PRJEB57691 accession code.
