# Supplementary material for: Transcriptome‐wide gene expression outlier analysis pinpoints therapeutic vulnerabilities in colorectal cancer
Source: Mol Oncol. 2024 Mar 11;18(6):1460–85. doi: 10.1002/1878-0261.13622 (PMC11161737; doi:10.1002/1878-0261.13622)
Supplement: Supplementary file 1 — Fig. S1. Gene expression‐based hierarchical clustering of 226 colorectal cancer (CRC) cell lines. Fig. S2. Correspondences between consensus molecular subtypes (CMS) and CRC intrinsic subtypes (CRIS) identified in 226 colorectal cancer (CRC) cell lines. Fig. S3. AnnotaGon of the CpG island methylator phenotype (CIMP) in 226 colorectal cancer (CRC) cell lines. Fig. S4. Identification of extreme negative outliers for the CNKSR1 gene. Fig. S5. Outlier burden values are associated with molecular features of colorectal cancer (CRC) cell lines. Fig. S6. Promoter hypermethylation and microsatellite instability in MLH1 extreme negative outliers. Fig. S7. Expression rank plots generated by Outlier in RNA‐seq Finder (OUTRIDER) for a selection of relevant genes. Fig. S8. Promoter hypermethylation in RAD51C extreme negative outliers. Fig. S9. Genomic position of analyzed CpG sites in transcript promoters with respect to CpG islands. Fig. S10. Expression levels of genes involved in somatic fusion transcripts associated with gene overexpression in extreme positive outliers. Fig. S11. CDKN2A and CDKN2B deletion is associated with very low or zero expression values in colorectal cancer (CRC) cell lines. Fig. S12. AGI‐24512 and MRTX1719 screening in MTAP‐deleted and wild‐type colorectal cancer (CRC) cell lines. Fig. S13. Positive and negative outliers found in The Cancer Genome Atlas (TCGA) dataset are usually less prominent than those found in the colorectal cancer (CRC) cell line dataset. Table S1. Annotation of the 226 colorectal cancer (CRC) cell lines. Table S2. Short tandem repeats (STR) profiles of the 226 colorectal cancer (CRC) cell lines considering 16 different loci. Table S3. Atlas of colorectal cancer (CRC) extreme gene expression outliers. Table S4. Differential methylation analysis for genes for which at least two colorectal cancer (CRC) cell lines were found as extreme positive (first sheet) or negative (second sheet) outliers. Table S5. Enrichment of samples that ar [file MOL2-18-1460-s001.zip › mol213622-sup-0001-FigsS1-S13.pdf]

Figure S1

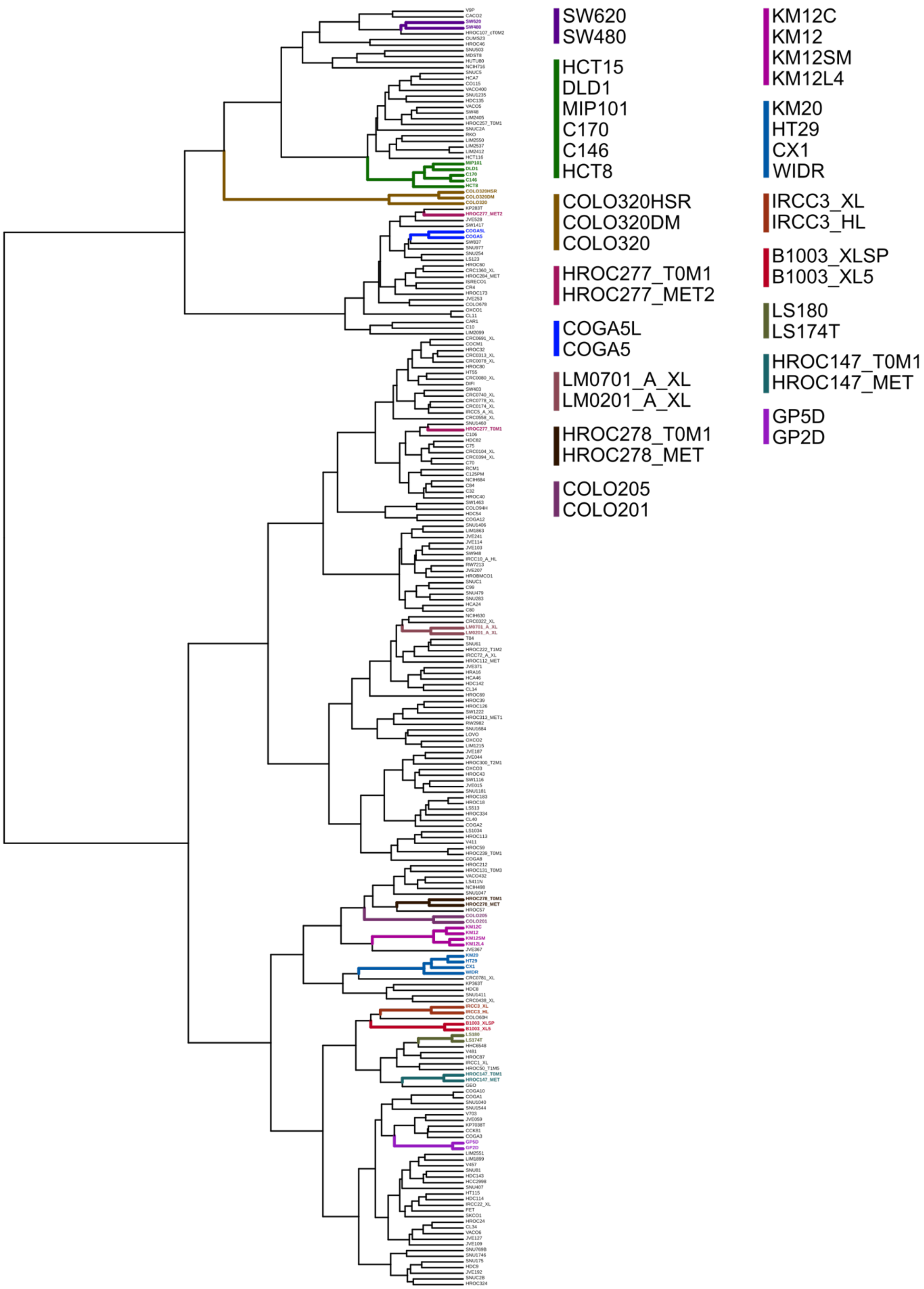
SW620
SW480
HCT15
DLD1
MIP101
C170
C146
HCT8
COLO320HSR
COLO320DM
COLO320
HROC277_T0M1
HROC277_MET2
COGA5L
COGA5
LM0701_A_XL
LM0201_A_XL
HROC278_T0M1
HROC278_MET
COLO205
COLO201
KM12C
KM12
KM12SM
KM12L4
KM20
HT29
CX1
WIDR
IRCC3_XL
IRCC3_HL
B1003_XLSP
B1003_XL5
LS180
LS174T
HROC147_T0M1
HROC147_MET
GP5D
GP2D

Figure S2

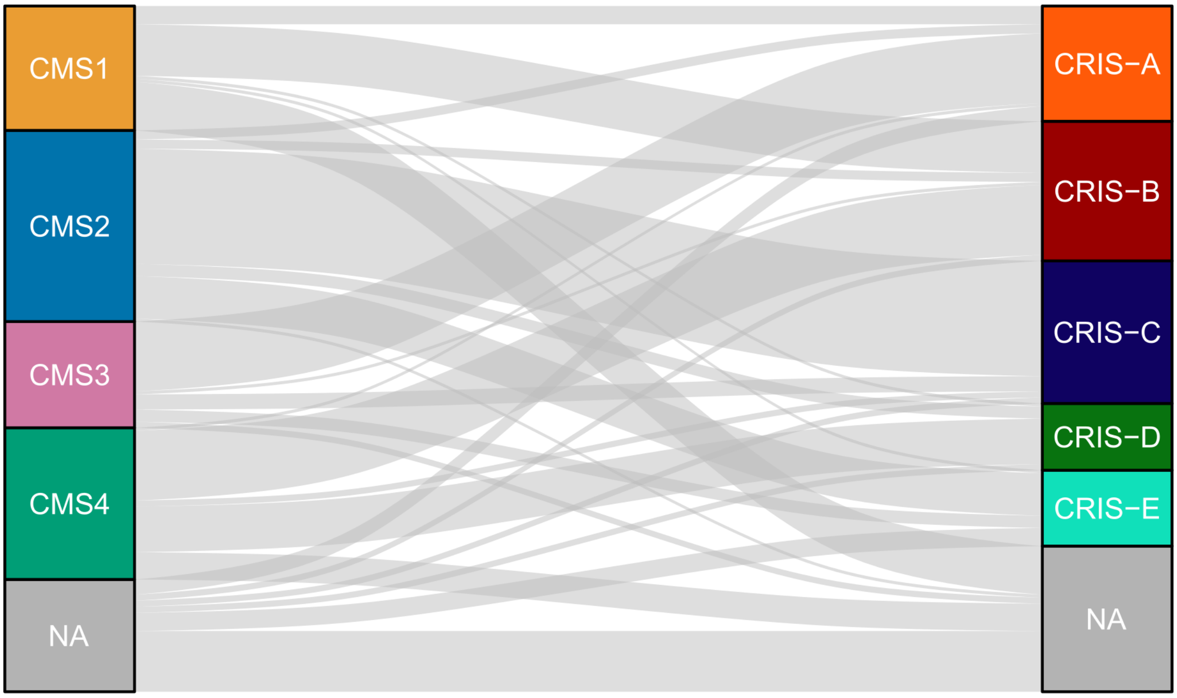

Figure S3

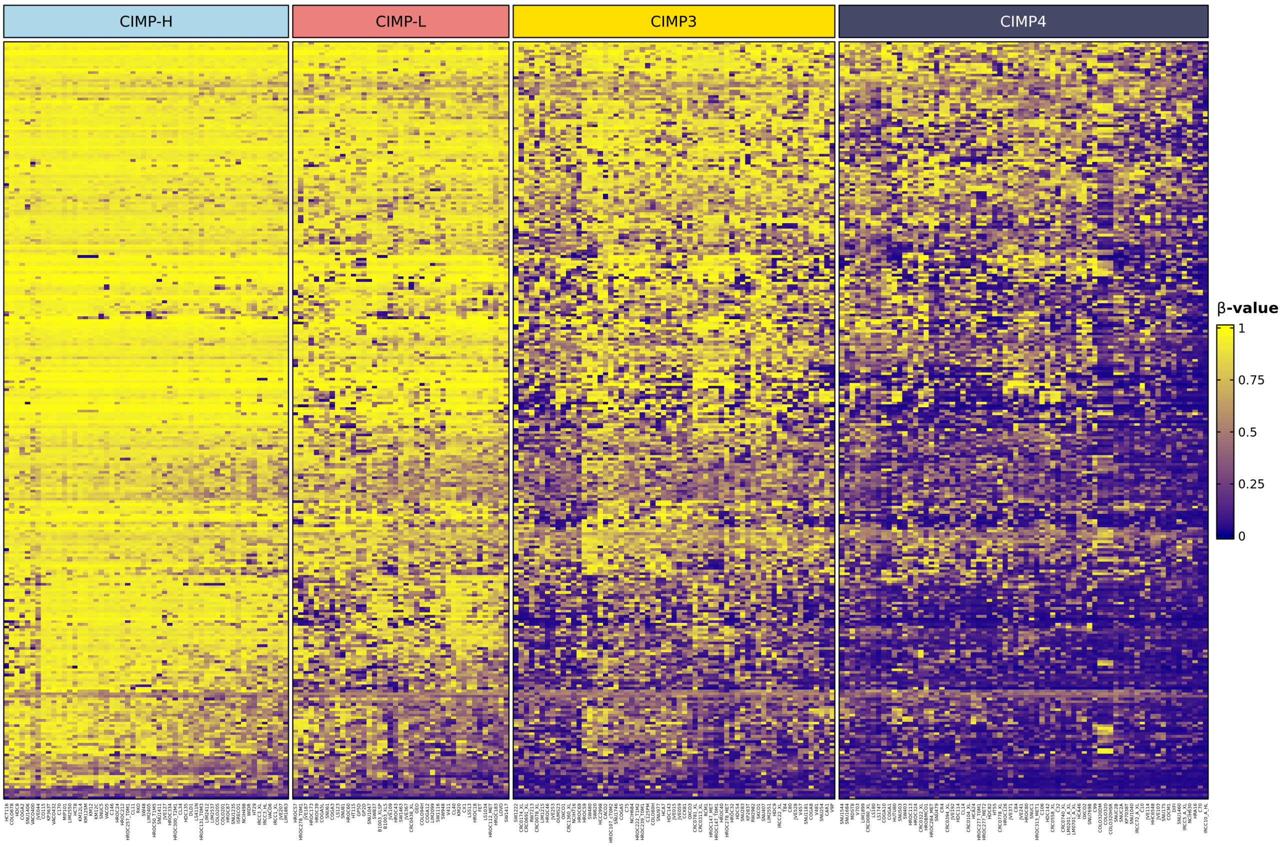

Figure S4

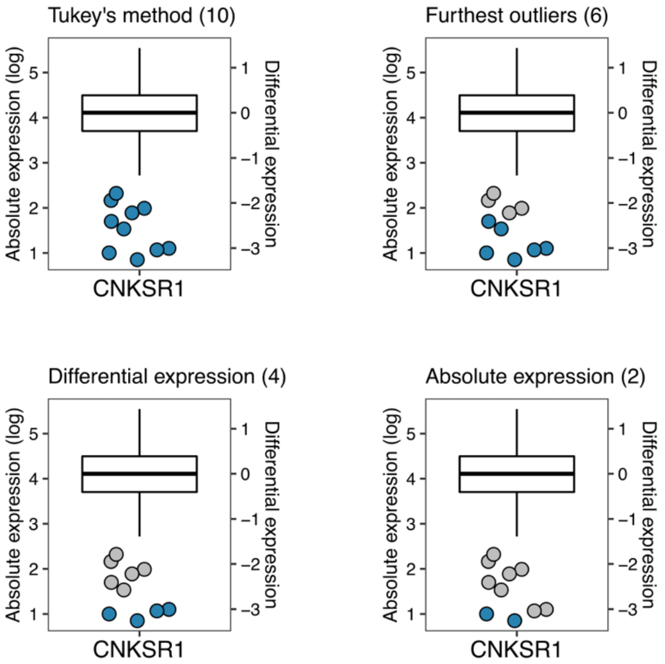

Tukey's method (10)
Furthest outliers (6)
Differential expression (4)
Absolute expression (2)
Absolute expression (log)
Differential expression
CNKSR1

Figure S5

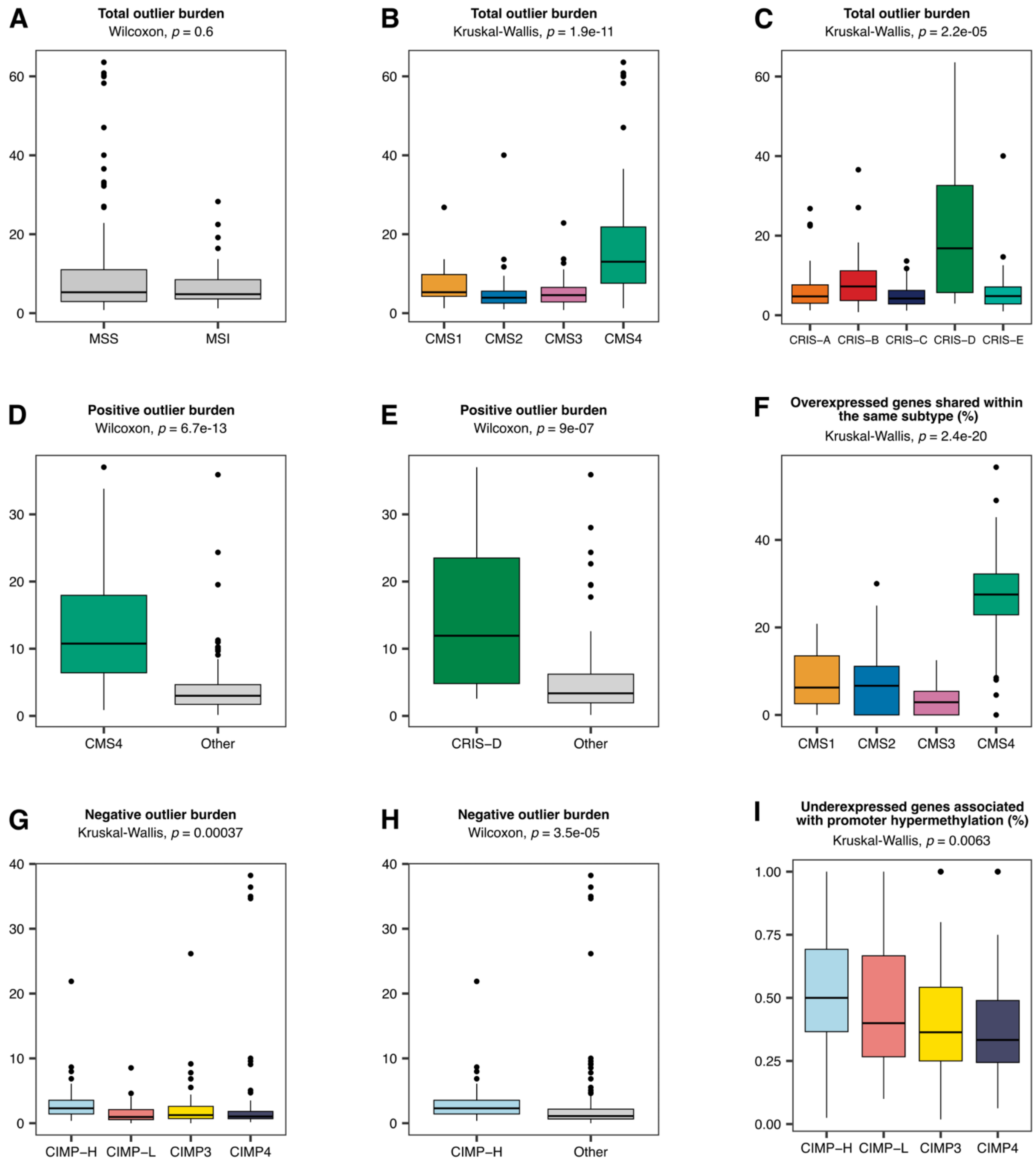
A
Total outlier burden
Wilcoxon, p = 0.6
MSS
MSI
B
Total outlier burden
Kruskal-Wallis, p = 1.9e-11
CMS1
CMS2
CMS3
CMS4
C
Total outlier burden
Kruskal-Wallis, p = 2.2e-05
CRIS−A
CRIS−B
CRIS−C
CRIS−D
CRIS−E
D
Positive outlier burden
Wilcoxon, p = 6.7e-13
CMS4
Other
E
Positive outlier burden
Wilcoxon, p = 9e-07
CRIS−D
Other
F
Overexpressed genes shared within the same subtype (%)
Kruskal-Wallis, p = 2.4e-20
CMS1
CMS2
CMS3
CMS4
G
Negative outlier burden
Kruskal-Wallis, p = 0.00037
CIMP−H
CIMP−L
CIMP3
CIMP4
H
Negative outlier burden
Wilcoxon, p = 3.5e-05
CIMP−H
Other
I
Underexpressed genes associated with promoter hypermethylation (%)
Kruskal-Wallis, p = 0.0063
CIMP−H
CIMP−L
CIMP3
CIMP4

Figure S6

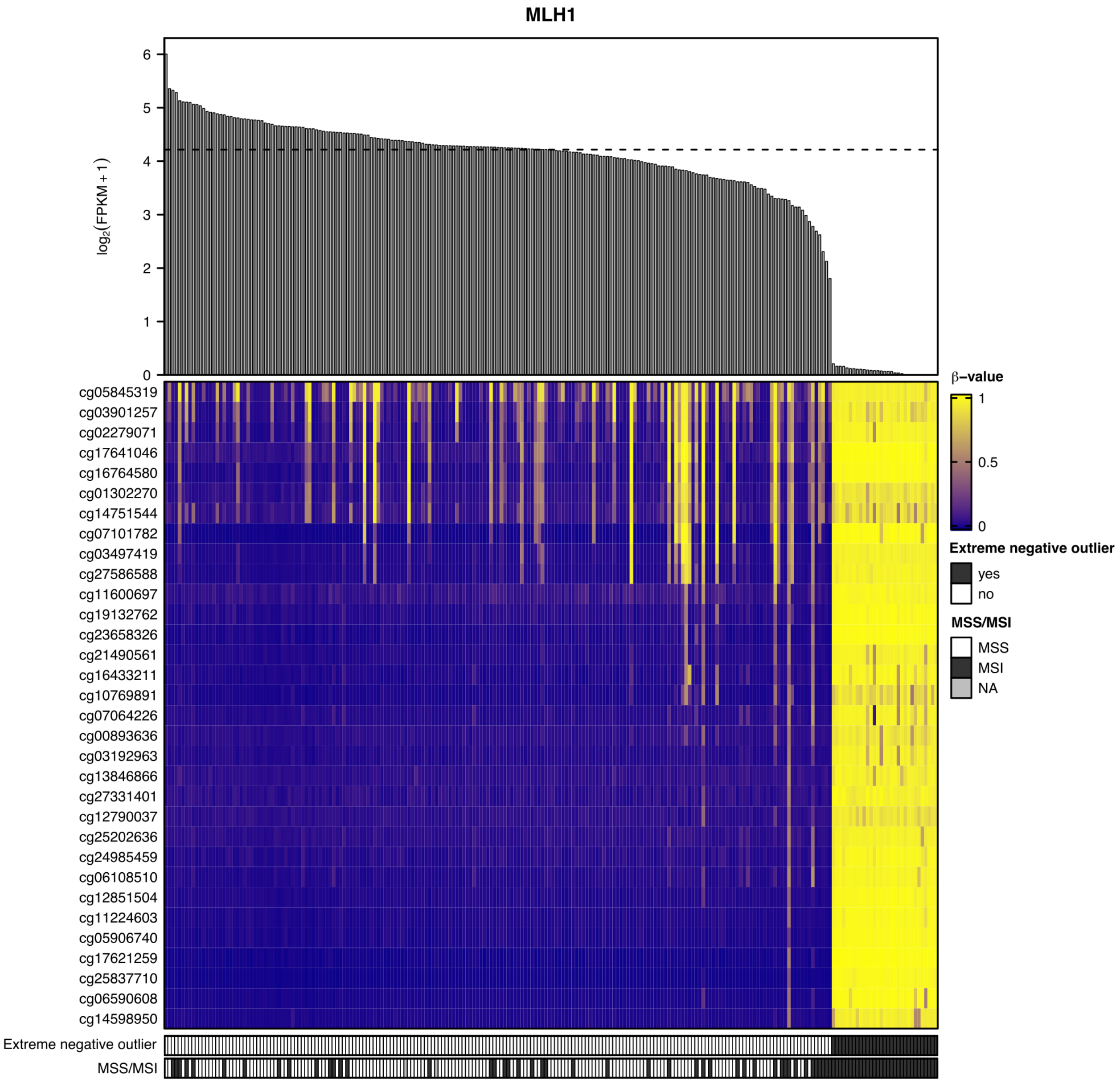
MLH1
log2(FPKM+1)
0
1
2
3
4
5
6
cg05845319
cg03901257
cg02279071
cg17641046
cg16764580
cg01302270
cg14751544
cg07101782
cg03497419
cg27586588
cg11600697
cg19132762
cg23658326
cg21490561
cg16433211
cg10769891
cg07064226
cg00893636
cg03192963
cg13846866
cg27331401
cg12790037
cg25202636
cg24985459
cg06108510
cg12851504
cg11224603
cg05906740
cg17621259
cg25837710
cg06590608
cg14598950
Extreme negative outlier
MSS/MSI
β−value
1
0.5
0
Extreme negative outlier
yes
no
MSS/MSI
MSS
MSI
NA

Figure S7

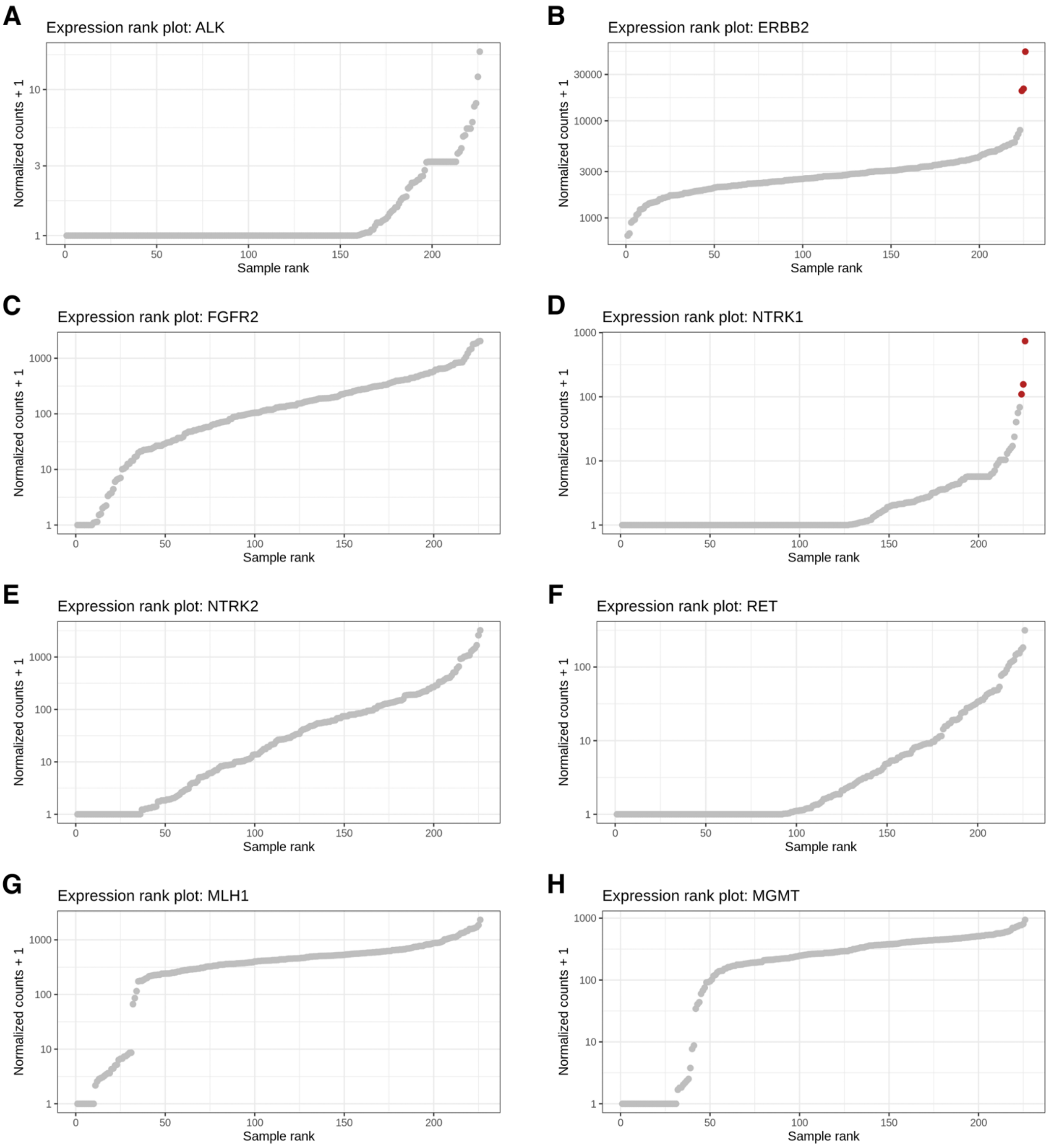
A
Expression rank plot: ALK
Normalized counts + 1
1
3
10
0
50
100
150
200
Sample rank
B
Expression rank plot: ERBB2
Normalized counts + 1
1000
3000
10000
30000
0
50
100
150
200
Sample rank
C
Expression rank plot: FGFR2
Normalized counts + 1
1
10
100
1000
0
50
100
150
200
Sample rank
D
Expression rank plot: NTRK1
Normalized counts + 1
1
10
100
1000
0
50
100
150
200
Sample rank
E
Expression rank plot: NTRK2
Normalized counts + 1
1
10
100
1000
0
50
100
150
200
Sample rank
F
Expression rank plot: RET
Normalized counts + 1
1
10
100
0
50
100
150
200
Sample rank
G
Expression rank plot: MLH1
Normalized counts + 1
1
10
100
1000
0
50
100
150
200
Sample rank
H
Expression rank plot: MGMT
Normalized counts + 1
1
10
100
1000
0
50
100
150
200
Sample rank

Figure S8

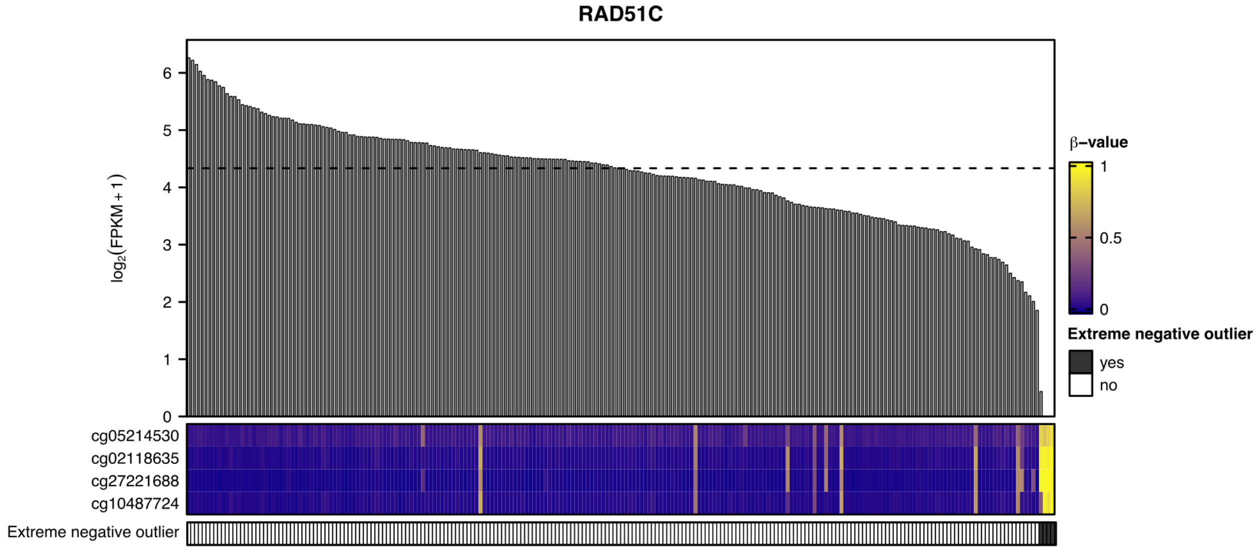

RAD51C
log2(FPKM + 1)
6
5
4
3
2
1
0
cg05214530
cg02118635
cg27221688
cg10487724
Extreme negative outlier
β−value
1
0.5
0
Extreme negative outlier
yes
no

Figure S9

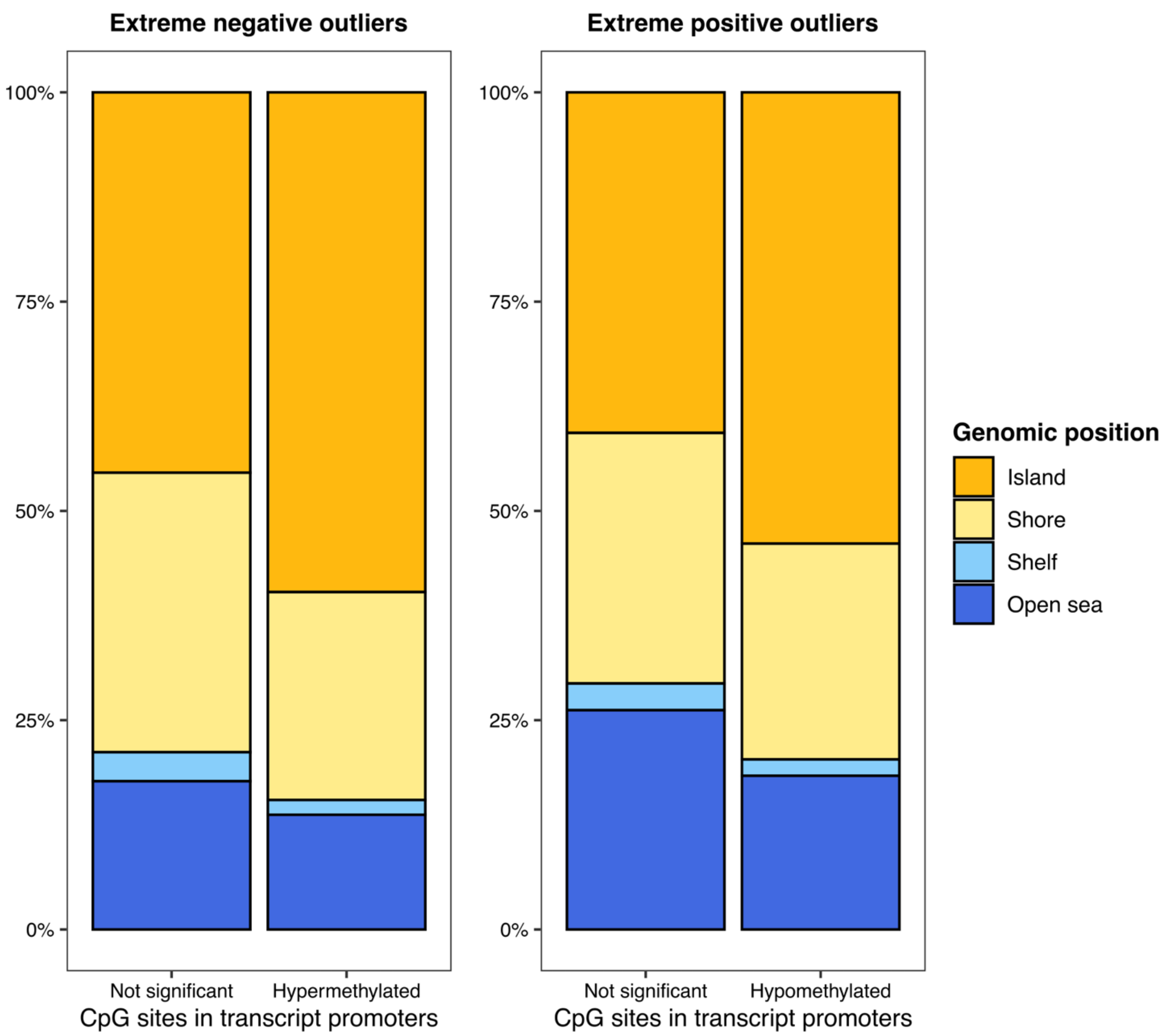
Extreme negative outliers
Extreme positive outliers
100%
75%
50%
25%
0%
Not significant
Hypermethylated
Hypomethylated
CpG sites in transcript promoters
Genomic position
Island
Shore
Shelf
Open sea

Figure S10

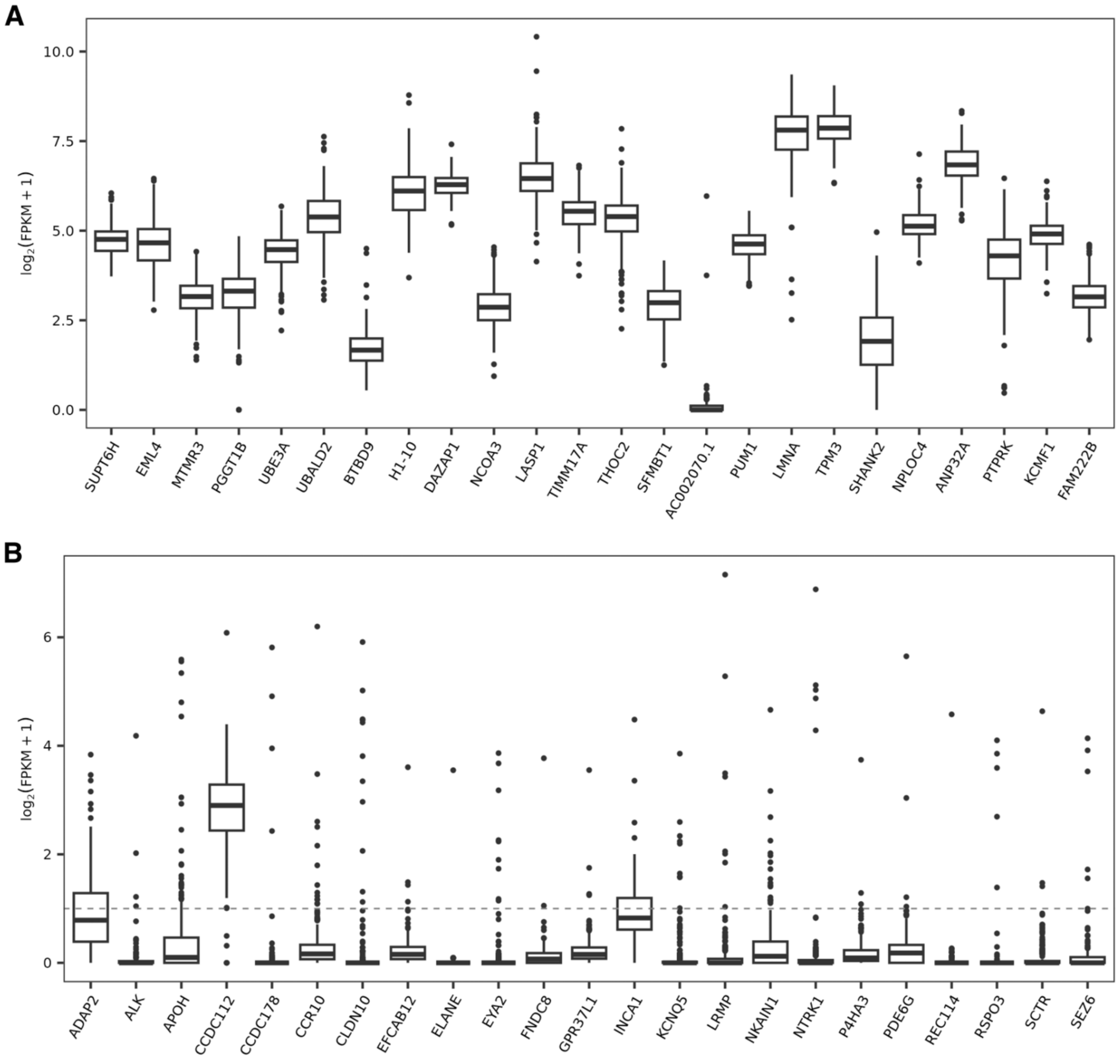
A
10.0
7.5
5.0
2.5
0.0
$\log_2(\mathrm{FPKM}+1)$
SUPT6H
EML4
MTMR3
PGGT1B
UBE3A
UBALD2
BTBD9
H1-10
DAZAP1
NCOA3
LASP1
TIMM17A
THOC2
SFMBT1
AC002070.1
PUM1
LMNA
TPM3
SHANK2
NPLOC4
ANP32A
PTPRK
KCMF1
FAM222B
B
6
4
2
0
$\log_2(\mathrm{FPKM}+1)$
ADAP2
ALK
APOH
CCDC112
CCDC178
CCR10
CLDN10
EFCAB12
ELANE
EYA2
FNDC8
GPR37L1
INCA1
KCNQ5
LRMP
NKAIN1
NTRK1
P4HA3
PDE6G
REC114
RSPO3
SCTR
SEZ6

Figure S11

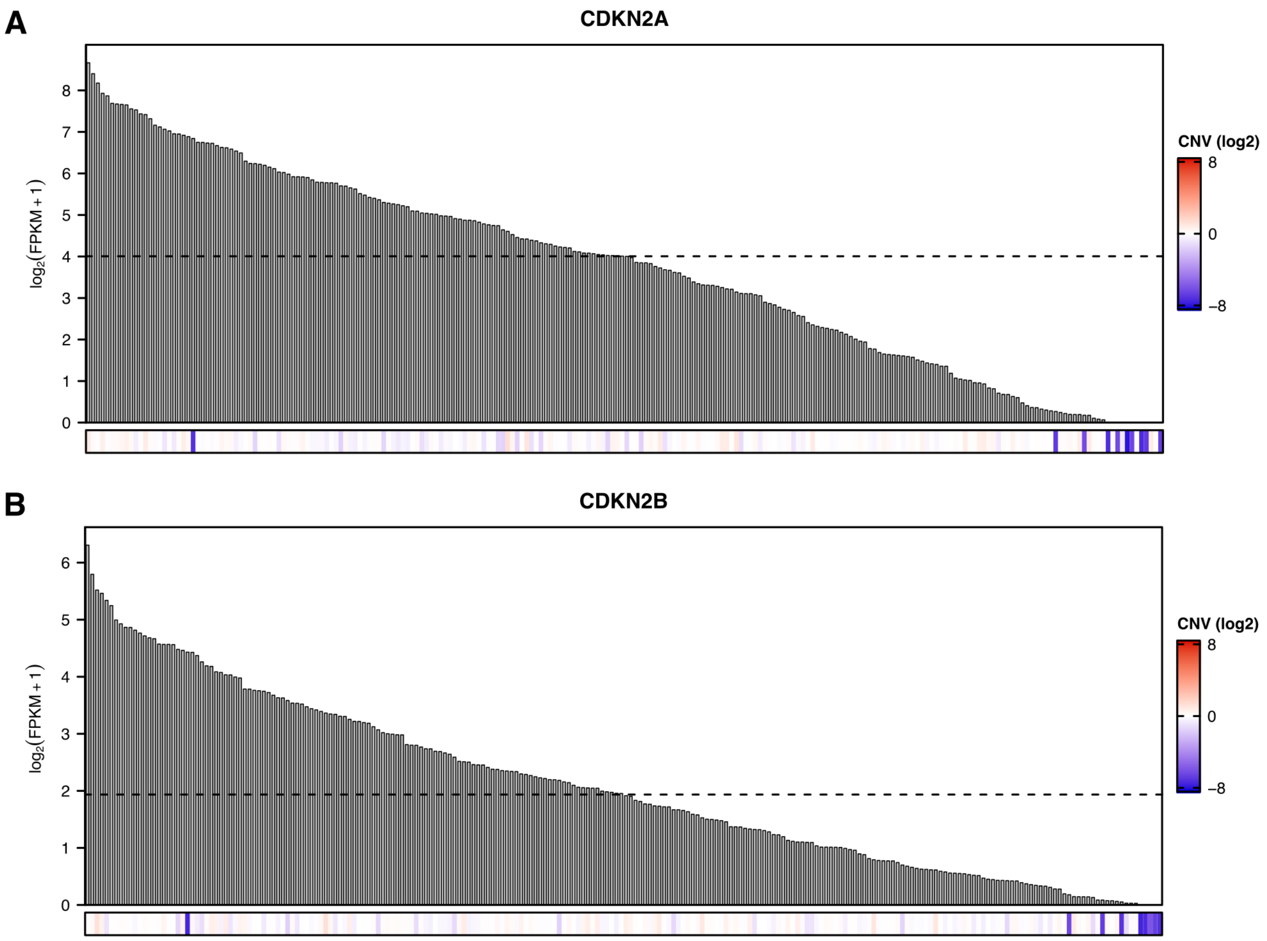
A
CDKN2A
log2(FPKM + 1)
8
7
6
5
4
3
2
1
0
CNV (log2)
8
0
−8
B
CDKN2B
log2(FPKM + 1)
6
5
4
3
2
1
0
CNV (log2)
8
0
−8

Figure S12

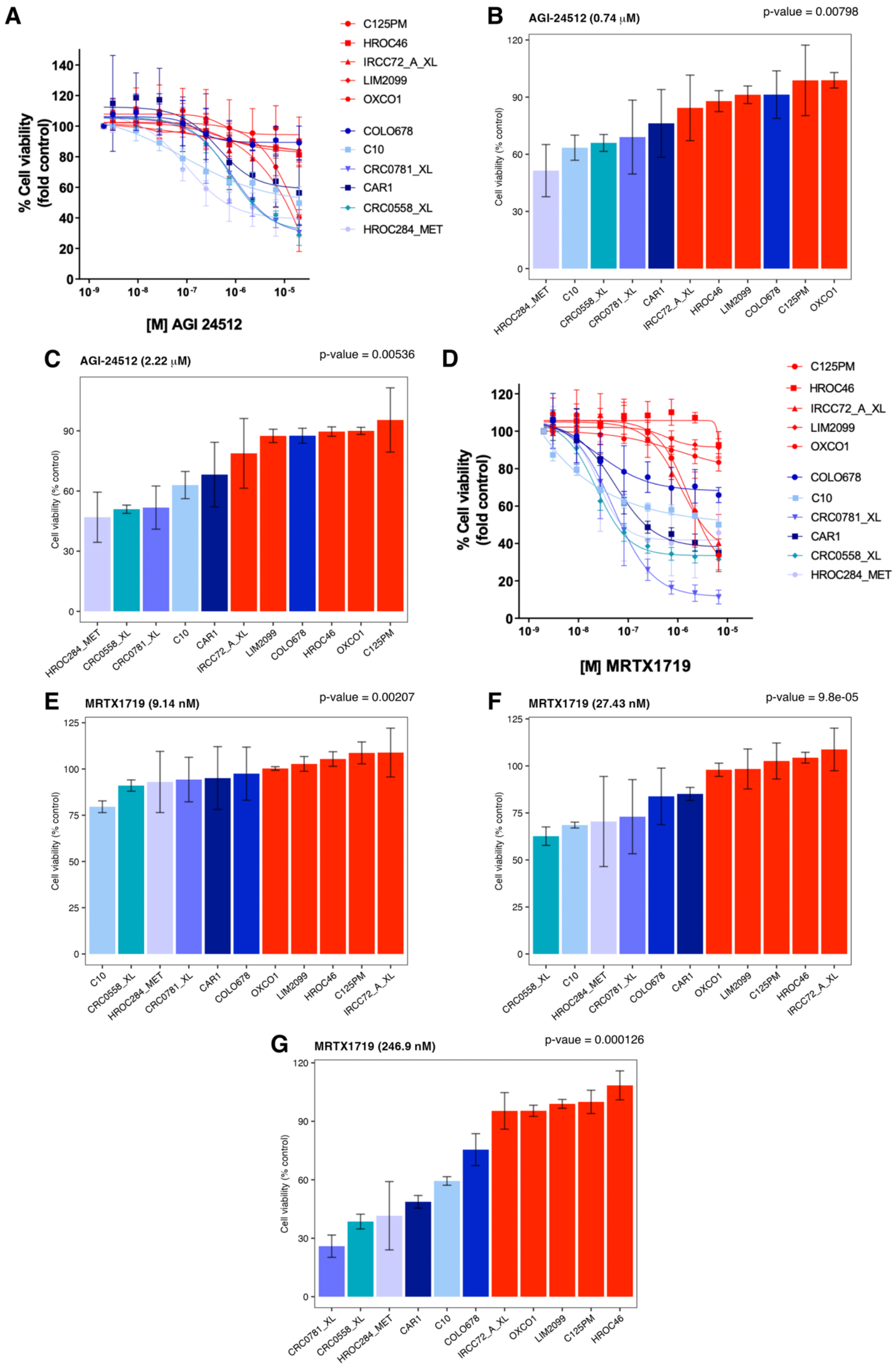
A
% Cell viability
(fold control)
[M] AGI 24512
C125PM
HROC46
IRCC72_A_XL
LIM2099
OXCO1
COLO678
C10
CRC0781_XL
CAR1
CRC0558_XL
HROC284_MET
B
AGI-24512 (0.74 μM)
p-value = 0.00798
Cell viability (% control)
HROC284_MET
C10
CRC0558_XL
CRC0781_XL
CAR1
IRCC72_A_XL
HROC46
LIM2099
COLO678
C125PM
OXCO1
C
AGI-24512 (2.22 μM)
p-value = 0.00536
Cell viability (% control)
HROC284_MET
CRC0558_XL
CRC0781_XL
C10
CAR1
IRCC72_A_XL
LIM2099
COLO678
HROC46
OXCO1
C125PM
D
% Cell viability
(fold control)
[M] MRTX1719
C125PM
HROC46
IRCC72_A_XL
LIM2099
OXCO1
COLO678
C10
CRC0781_XL
CAR1
CRC0558_XL
HROC284_MET
E
MRTX1719 (9.14 nM)
p-value = 0.00207
Cell viability (% control)
C10
CRC0558_XL
HROC284_MET
CRC0781_XL
CAR1
COLO678
OXCO1
LIM2099
HROC46
C125PM
IRCC72_A_XL
F
MRTX1719 (27.43 nM)
p-value = 9.8e-05
Cell viability (% control)
CRC0558_XL
C10
HROC284_MET
CRC0781_XL
COLO678
CAR1
OXCO1
LIM2099
C125PM
HROC46
IRCC72_A_XL
G
MRTX1719 (246.9 nM)
p-vaue = 0.000126
Cell viability (% control)
CRC0781_XL
CRC0558_XL
HROC284_MET
CAR1
C10
COLO678
IRCC72_A_XL
OXCO1
LIM2099
C125PM
HROC46

Figure S13

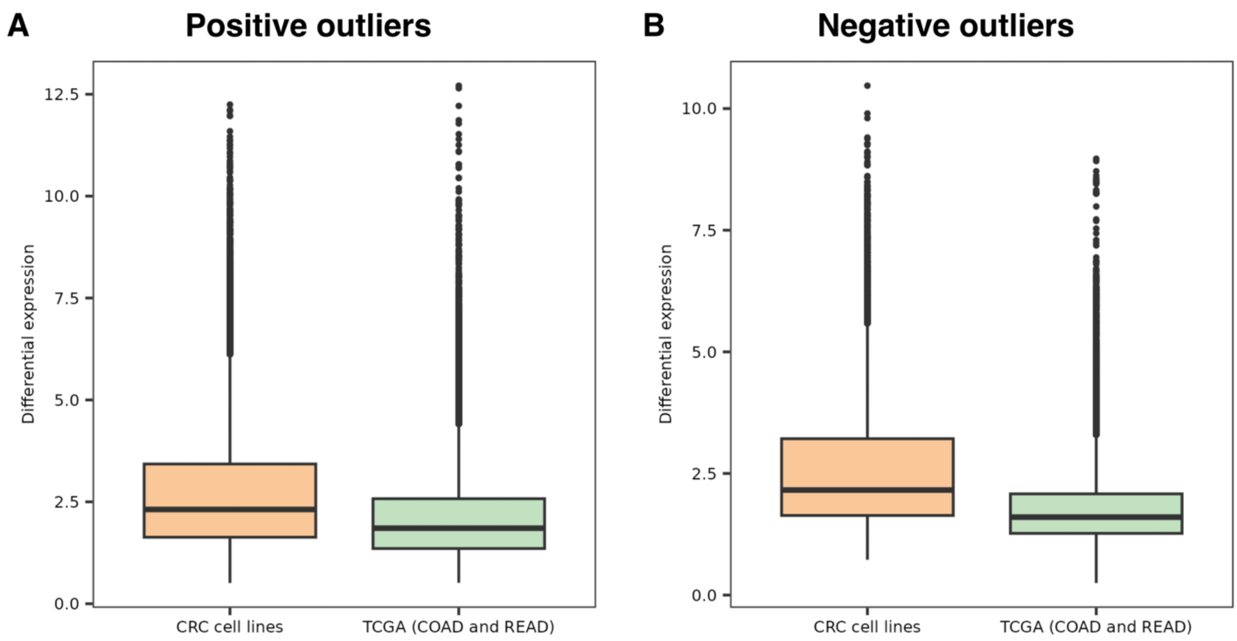
A
Positive outliers
B
Negative outliers
Differential expression
12.5
10.0
7.5
5.0
2.5
0.0
CRC cell lines
TCGA (COAD and READ)
Differential expression
10.0
7.5
5.0
2.5
0.0
CRC cell lines
TCGA (COAD and READ)
