# Supplementary material for: Transcriptome‐wide gene expression outlier analysis pinpoints therapeutic vulnerabilities in colorectal cancer
Source: Mol Oncol. 2024 Mar 11;18(6):1460–85. doi: 10.1002/1878-0261.13622 (PMC11161737; doi:10.1002/1878-0261.13622)
Supplement: Supplementary file 1 — Fig. S1. Gene expression‐based hierarchical clustering of 226 colorectal cancer (CRC) cell lines. Fig. S2. Correspondences between consensus molecular subtypes (CMS) and CRC intrinsic subtypes (CRIS) identified in 226 colorectal cancer (CRC) cell lines. Fig. S3. AnnotaGon of the CpG island methylator phenotype (CIMP) in 226 colorectal cancer (CRC) cell lines. Fig. S4. Identification of extreme negative outliers for the CNKSR1 gene. Fig. S5. Outlier burden values are associated with molecular features of colorectal cancer (CRC) cell lines. Fig. S6. Promoter hypermethylation and microsatellite instability in MLH1 extreme negative outliers. Fig. S7. Expression rank plots generated by Outlier in RNA‐seq Finder (OUTRIDER) for a selection of relevant genes. Fig. S8. Promoter hypermethylation in RAD51C extreme negative outliers. Fig. S9. Genomic position of analyzed CpG sites in transcript promoters with respect to CpG islands. Fig. S10. Expression levels of genes involved in somatic fusion transcripts associated with gene overexpression in extreme positive outliers. Fig. S11. CDKN2A and CDKN2B deletion is associated with very low or zero expression values in colorectal cancer (CRC) cell lines. Fig. S12. AGI‐24512 and MRTX1719 screening in MTAP‐deleted and wild‐type colorectal cancer (CRC) cell lines. Fig. S13. Positive and negative outliers found in The Cancer Genome Atlas (TCGA) dataset are usually less prominent than those found in the colorectal cancer (CRC) cell line dataset. Table S1. Annotation of the 226 colorectal cancer (CRC) cell lines. Table S2. Short tandem repeats (STR) profiles of the 226 colorectal cancer (CRC) cell lines considering 16 different loci. Table S3. Atlas of colorectal cancer (CRC) extreme gene expression outliers. Table S4. Differential methylation analysis for genes for which at least two colorectal cancer (CRC) cell lines were found as extreme positive (first sheet) or negative (second sheet) outliers. Table S5. Enrichment of samples that ar [file MOL2-18-1460-s001.zip › Supplementaty files-Legends.pdf]

Supplementary figures

Figure S1: Gene expression-based hierarchical clustering of 226 colorectal cancer (CRC) cell lines. Dendrogram depicting the hierarchical clustering of 226 CRC cell lines based on their gene expression profiles. Groups of genetically identical cell lines, i.e., cell lines with same short tandem repeats (STR) profile, that derived from a same individual are highlighted in different colors and their names are reported on the right in the order by which they occur in the dendrogram, from top to bottom.

Figure S2: Correspondences between consensus molecular subtypes (CMS) and CRC intrinsic subtypes (CRIS) identified in 226 colorectal cancer (CRC) cell lines. Alluvial plot depicting correspondences between CMS and CRIS gene expression- based subtypes identified in 226 CRC cell lines. In both classifications, CRC cell lines that cannot be confidently assigned to a single subtype (FDR>5%) were labelled as NA (not available).

Figure S3: Annotation of the CpG island methylator phenotype (CIMP) in 226 colorectal cancer (CRC) cell lines. Heatmap depicting DNA methylation levels of 289 CIMP associated probes in 226 CRC cell lines. DNA methylation levels were measured as β-values and are represented using a colour scale from dark blue (low DNA methylation level) to yellow (high DNA methylation level). DNA methylation-based subtypes were identified through unsupervised clustering and they were associated to different CIMP classes based on their median DNA methylation level (CIMP-H, CIMP-L, CIMP3 and CIMP4, from high to low median DNA methylation level), as reported above the heatmap. Samples are split according to CIMP classes and, in each slice, they are arranged based on hierarchical clustering done using the euclidean distance metric and average linkage. The probes are arranged based on hierarchical clustering done using the euclidean distance metric and average linkage.

Figure S4: Identification of extreme negative outliers for the *CNKSR1* gene. Each boxplot shows the distribution of CNKSR1 expression values. Blue dots are samples selected as negative outliers after each step of the pipeline. Their number is also reported between brackets in the title of each subpanel.

Figure S5: Outlier burden values are associated with molecular features of colorectal cancer (CRC) cell lines. (A) Boxplots showing the distribution of total outlier burden values in samples stratified according to the microsatellite status defined as microsatellite stability (MSS) or instability (MSI). (B) Boxplots showing the distribution of total outlier burden values in samples stratified according to consensus molecular subtypes (CMS). (C) Boxplots showing the distribution of total outlier burden values in samples stratified according to CRC intrinsic subtypes (CRIS). (D) Boxplots showing the distribution of positive outlier burden values in samples stratified according to whether they belong to the CMS4 subtype or to other CMS subtypes. (E) Boxplots showing the distribution of positive outlier burden values in samples stratified according to whether they belong to the CRIS-D subtype or to other CRIS subtypes. (F) Boxplots showing the distribution of the fraction of overexpressed genes whose overexpression only occurs within the same subtype in samples stratified according to the CMS transcriptional classification. (G) Boxplots showing the distribution of negative outlier burden values in samples stratified according to CpG island methylator phenotype (CIMP). (H) Boxplots showing the distribution of negative outlier burden values in samples stratified according to whether they belong to the CIMP-H subtype or to other DNA methylation-based subtypes. (I) Boxplots showing the distribution of the fraction of underexpressed genes whose silencing is

associated with promoter hypermethylation in samples stratified according to the CIMP classification. In each boxplot the box corresponds to the interquartile range (IQR) and the median is represented by the center line. Whiskers maximally extend to 1.5 × IQR and outliers are shown. P-values that are shown above each graph were calculated by a Wilcoxon test when two sample groups are present, or by a Kruskal-Wallis test when multiple sample groups exists.

Figure S6: Promoter hypermethylation and microsatellite instability in *MLH1* extreme negative outliers. Heatmap depicting DNA methylation levels of promoter probes that were found differentially methylated between *MLH1* extreme negative outliers and other samples. DNA methylation levels were measured as β-values and are represented using a colour scale from dark blue (low DNA methylation level) to yellow (high DNA methylation level). *MLH1* expression profile is shown above the heatmap and sample ordering is the same in the two graphs, from high to low *MLH1* expression levels. Annotation bars below the heatmap indicate – from top to bottom – samples that were scored as *MLH1* extreme negative outliers and the microsatellite status of each sample defined as microsatellite stability (MSS) or microsatellite instability (MSI).

Figure S7: Expression rank plots generated by Outlier in RNA-Seq Finder (OUTRIDER) for a selection of relevant genes. In each panel, the controlled counts of a single gene are shown in ascending order. Points corresponding to samples recognized as outliers by OUTRIDER are red.

Figure S8: Promoter hypermethylation in *RAD51C* extreme negative outliers. Heatmap depicting DNA methylation levels of promoter probes that were found differentially methylated between *RAD51C* extreme negative outliers and other samples. DNA methylation levels were measured as β-values and are represented using a color scale from dark blue (low DNA methylation level) to yellow (high DNA methylation level). *RAD51C* expression profile is shown above the heatmap and sample ordering is the same in the two graphs, from high to low *RAD51C* expression levels. The annotation bar below the heatmap indicates samples that were pinpointed as *RAD51C* extreme negative outliers.

Figure S9: Genomic position of analysed CpG sites in transcript promoters with respect to CpG islands. The genomic position of CpG sites within transcript promoters that were tested for differential methylation was determined with respect to CpG islands. Islands, shores, shelfs and open sea were defined as detailed in the method section. In the left panel, stacked bar graph depicting the proportion of analysed CpG sites in different genomic regions for both not significant CpG sites and those that are significantly hypermethylated in extreme negative outliers. In the right panel, stacked bar graph depicting the proportion of analysed CpG sites in different genomic regions for both not significant CpG sites and those that are significantly hypomethylated in extreme positive outliers.

Figure S10: Expression levels of genes involved in somatic fusion transcripts associated with gene overexpression in extreme positive outliers. Boxplots depicting the distribution of expression values of genes that are involved as 5' (A) or 3' (B) partner genes in somatic fusions transcripts associated with gene overexpression in extreme positive outliers. The box corresponds to the interquartile range (IQR) and the median is represented by the center line. Whiskers maximally extend to 1.5 × IQR and outliers are shown. In (B) the dashed gray line corresponds to the threshold used to identify the silenced genes (median FPKM < 1).

Figure S11: *CDKN2A* and *CDKN2B* deletion is associated with very low or zero expression values in colorectal cancer (CRC) cell lines. (A) Heatmap depicting log2-transformed copy number variation (CNV) values of *CDKN2A* in 226 CRC cell lines. *CDKN2A* expression profile is shown above the heatmap and sample ordering is the same in the two graphs, from high to low *CDKN2A* expression levels. (B) Heatmap depicting log2-transformed copy number variation (CNV) values of *CDKN2B* in 226 CRC cell lines. *CDKN2B* expression profiles are shown above the heatmap and sample ordering is the same in the two graphs, from high to low *CDKN2B* expression levels. In both cases, log2-transformed CNV values are represented using a colour scale from red (gene amplification) to blue (gene deletion) and white indicates copy number neutrality.

Figure S12: AGI-24512 and MRTX1719 screening in *MTAP*-deleted and wild-type colorectal cancer (CRC) cell lines. (A) Cell viability was assessed by measuring ATP content after 7 days of treatment with increasing concentration of AGI-24512. Red lines are negative controls in which the *MTAP* gene is normally expressed. Cell viability measured for each treatment condition was normalized to viability of DMSO-treated cells. (B-C) Cell viability (% control cells treated with DMSO) measured in *MTAP*-deleted (blue bars) and wild-type (red bards) CRC cell lines after treatment with AGI-24512 at different concentrations. (D) Cell viability was assessed by measuring ATP content after 7 days of treatment with increasing concentration of MRTX1719. Red lines are negative controls in which the *MTAP* gene is normally expressed. Cell viability measured for each treatment condition was normalized to viability of DMSO-treated cells. (E-G) Cell viability (% control cells treated with DMSO) measured in *MTAP*-deleted (blue bars) and wild-type (red bards) CRC cell lines after treatment with MRTX1719 at different concentrations. In (B-C) and (E-G) the p-value obtained with one-way ANOVA test is reported for each treatment condition. Data represent mean ± SD of at least three independent biological replicates.

Figure S13: Positive and negative outliers found in The Cancer Genome Atlas (TCGA) dataset are usually less prominent than those found in the colorectal cancer (CRC) cell line dataset. Boxplots depicting the distribution of differential expression values (log2 fold change with respect to the median gene expression) after the selection of the furthest positive (A) or negative (B) outliers for each gene in the CRC cell line dataset or in the TCGA dataset. The box corresponds to the interquartile range (IQR) and the median is represented by the center line. Whiskers maximally extend to 1.5 × IQR and outliers are shown.

Supplementary tables

Table S1. Annotation of the 226 colorectal cancer (CRC) cell lines. For each cell line we report: (1) **CRC cell line name**; (2) **Alternative name**; (3) **Research Resource Identifiers (RRIDs)**, as available in the ExPASy Cellosaurus database; (4) **Source**: established in our lab from surgical samples or patient-derived xenografts (PDXs), obtained through a collaboration, purchased from an international cell line bank; (5) **Supplier ID**: detail about the cell line origin, i.e., the Professor with whom the collaboration was established or name of the international cell line bank; (6) **Microsatellite status**: microsatellite stability (MSS) or instability (MSI); (7-11) ***KRAS, NRAS, BRAF, APC, TP53*****:** mutational status for these genes as defined in the main text; (12) **CMS**: consensus molecular subtypes (CMS1, CMS2, CMS3 or CMS4). Cell lines that cannot be confidently assigned to a single subtype (FDR>5%) were labeled as NA (not available); (13) **CRIS**: colorectal cancer intrinsic subtypes (CRIS-A, CRIS-B, CRIS-C, CRIS-D or CRIS-E). Cell lines that cannot be confidently assigned to a single subtype (FDR>5%) were labeled as NA (not available); (14) **CIMP**: status for the CpG island methylator phenotype

(CIMP-H, CIMP-L, CIMP3 and CIMP4); (15) **References**: publications in which additional information about each cell line can be found.

Table S2. Short tandem repeats (STR) profiles of the 226 colorectal cancer (CRC) cell lines considering 16 different loci.

Table S3. Atlas of colorectal cancer (CRC) extreme gene expression outliers. The first sheet includes a summary of how the number of genes and samples changes along the different steps of the transcriptome-wide gene expression outlier analysis that we performed in CRC cells. Then, for each extreme positive (second sheet) or negative (third sheet) outlier we report: (1) **Gene ID**: Ensembl gene ID of the gene for which an extreme outlier was found; (2) **Gene name**: gene symbol of the gene for which an extreme outlier was found; (3) **Sample**: name of the CRC cell line identified as extreme outlier for the indicated gene; (4) **Absolute expression**: FPKM value of the gene in the outlier sample; (5) **Differential expression**: log2 fold change with respect to the median gene level; (6) **Enzyme gene**: whether the gene encodes for an enzyme (YES) or not (NO); (7) **Kinase gene**: whether the gene encodes for a kinase (YES) or not (NO); (8) **Target development level (TDL)**: for enzyme genes we report the TDL category (Tclin, Tchem, Tbio and Tdark) as defined by Illuminating the Druggable Genome (IDG); (9) **Genetic feature**: genetic alteration associated with the outlier expression value when known (gene amplification, gene deletion or gene fusion).

Table S4. Differential methylation analysis for genes for which at least two colorectal cancer (CRC) cell lines were found as extreme positive (first sheet) or negative (second sheet) outliers. For each tested promoter probe we report: (1) **Gene ID**: Ensembl gene ID of the promoter target gene; (2) **Gene name**: gene symbol of the promoter target gene; (3) **Probe**: ID of the methylation probe; (4) **Median outliers**: median ß-value in outlier samples; (5) **Median others**: medium ß-value in non-outlier samples; (6) **p-value**: Wilcoxon test p-value for differential methylation between outlier and non- outlier samples; (7) **FDR**: False Discovery Rate; (8) **Genome segment**: genomic position of the CpG site targeted by the probe with respect to CpG islands (Islands, shores, shelves and open sea).

Table S5. Enrichment of samples that are positive for the CpG island methylator phenotype (CIMP) among the extreme negative outliers of a single gene. For each tested gene we report: (1) **Gene ID**: Ensembl gene ID; (2) **Gene name**: gene symbol; (3) **# outliers**: number of extreme negative outliers; (4-7) **CIMP-H, CIMP-L, CIMP3, CIMP4**: number of extreme negative outliers for each CIMP class; (8) **p-value**: hypergeometric test p-value; (9) **FDR**: False Discovery Rate.
